# A *Tac1*‐Expressing Brainstem Pathway Underlies the Pathogenesis of Trigeminal Neuralgia

**DOI:** 10.1002/advs.202516310

**Published:** 2026-03-13

**Authors:** Liting Sun, Jia‐Jia Wang, Xiang‐Yu Li, Xin‐Yi Lin, Juan Li, Qiu‐Tong Yu, Zi‐Han Wang, Xue‐Ping Gao, Lei Jin, Wei‐Ke Li, Tian‐Lin Cheng, Juan Deng

**Affiliations:** ^1^ Department of Anesthesiology Huadong Hospital State Key Laboratory of Medical Neurobiology Institute for Translational Brain Research MOE Frontiers Center for Brain Science Fudan University Shanghai China; ^2^ Lingang Laboratory Shanghai China

**Keywords:** caudal part of spinal trigeminal nucleus (Sp5C), CION, parabrachial nucleus (PBN), Sp5C projection neurons, Tac1 gene, trigeminal ganglion (TG), trigeminal neuralgia

## Abstract

Trigeminal neuralgia (TN) is widely recognized to be one of the most severe pain disorders, severely affects the life quality of patients. However, the neuronal mechanisms underlying TN pathogenesis remain largely unknown. Here, we identify a periphery‐to‐brain neural circuit that governs TN development in mice. Tachykinin 1 (*Tac1*)‐expressing parabrachial nucleus (PBN^Tac1^) neurons show heightened stimulus‐evoked responses, and chemogenetic inhibition of these neurons effectively prevents TN development. Furthermore, PBN^Tac1^ neurons receive projections from the caudal part of the spinal trigeminal nucleus (Sp5C), and PBN‐projecting Sp5C neurons are essential for TN‐induced pain hypersensitivity. Remarkably, PBN‐projecting Sp5C neurons predominantly express *Tac1*, and knockdown *Tac1* gene in these neurons significantly attenuates TN‐induced pain hypersensitivity. Through retrograde viral tracing and electrophysiological recordings, we demonstrate that *Tac1*‐expressing Sp5C neurons directly relay signals from the trigeminal ganglion (TG) to the PBN. Collectively, our findings unveil a critical TG‐Sp5C^Tac1^‐PBN^Tac1^ pathway that drives the TN pathogenesis.

## Graphical abstract

A critical TG‐Sp5C^Tac1^‐PBN^Tac1^ pathway drives trigeminal neuropathic pain (TNP). *Tac1*‐expressing parabrachial nucleus (PBN^Tac1^) neurons exhibit heightened responses to innocuous stimuli in TNP, and chemogenetic inhibition of these neurons effectively prevents TNP development. Furthermore, PBN^Tac1^ neurons receive projections from the Tac1‐expressing caudal part of spinal trigeminal nucleus (Sp5C ^Tac1^) which relay signals from the trigeminal ganglion (TG), and knockdown *Tac1* in Sp5C^Tac1^ neurons significantly attenuate TNP.

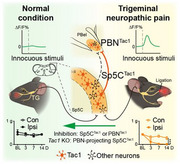

## 1 Introduction

Trigeminal neuralgia (TN) is characterized by recurrent, severe paroxysmal pain restricted to the trigeminal territory, often triggered by innocuous stimuli [1]. This condition profoundly impacts patients' psychological, physical, and social needs, such as touching the face, talking, eating, and drinking. Epidemiological studies estimate the lifetime prevalence of TN in the European population ranges from 0.16% to 0.3% [2]. Despite pharmacological intervention in 94% of cases, approximately two‐thirds of patients continue to suffer from moderate to severe pain [2]. This substantial clinical burden of TN underscores the clinical need to elucidate the underlying neural mechanisms and develop more effective therapeutic strategies.

Sensory neurons in the trigeminal ganglion (TG) are responsible for detecting and transmitting innocuous and noxious signals from the head and facial region to the central nervous system [3]. These neurons project to diverse brainstem nuclei and the cerebellum, forming extensive functional networks [4]. These projections play a pivotal role in mediating various physiological processes, including trigeminal reflexes (via the spinal cord), trigeminovisceral integration (via nucleus of the solitary tract, NTS), oral motor behaviors (via the caudal part of spinal trigeminal nucleus (Sp5C) and motor trigeminal nucleus) [5], orofacial reflexes (via the reticular formation), and emotion processing (via the parabrachial nucleus, PBN) [6]. Among all these brain areas, Sp5C emerges as a central hub for orofacial nociception. Analogous to spinal cord ascending pathways, Sp5C neurons further relay peripheral signals to the thalamus, hypothalamus, and PBN [7]. Notably, PBN‐projecting Sp5C neurons are activated by noxious stimulation from the corneal/dental nerve [8]. Functional magnetic resonance imaging (fMRI) studies in humans further corroborate these findings, demonstrating heightened activity in both Sp5C and the thalamus in TN patients during painful stimuli [9]. Despite the well‐established crucial role of Sp5C in orofacial nociception, the specific cellular mechanisms of Sp5C projection neurons in orofacial pain processing remain largely unknown. In the spinal cord, tracing and single‐cell sequencing studies have identified several molecular markers for projection neurons, including tachykinin 1 (*Tac1*), tachykinin receptor 1 (*Tacr1*), G‐protein‐coupled receptor 83 (*Grp83*), and LY6/PLAUR domain‐containing 1 (*Lypd1*) [10, 11, 12]. Among these, *Tacr1‐* and *Grp83*‐expressing neurons exhibit increased activity in response to noxious stimulation [12], and *Tac1*‐ and *Tacr1*‐expressing neurons are critically involved in somatic pain processing [13, 14, 15]. Intriguingly, some of these markers are also expressed in Sp5C [16, 17, 18]. However, their functional roles in the development of TN remain largely unexplored.

The PBN is one of the major downstream targets for Sp5C, and PBN‐projecting Sp5C neurons are activated by noxious orofacial stimulation [8]. In addition to the indirect pathway via Sp5C [2], the PBN also receives direct nociceptive inputs from the TG [6, 19, 20, 21]. Notably, inhibition of the direct TG‐PBN pathway significantly attenuates both facial nociception and pain‐related affective emotion [6]. Thus PBN integrates both direct and indirect trigeminal nociceptive signals to mediate orofacial pain processing. Moreover, the PBN has been implicated in the modulation of TN. In the chronic constriction injury of the infraorbital nerve (CION) model, PBN neurons exhibited hyperexcitability compared to those in sham‐operated rats [22]. Additionally, selective activation of the PBN‐ventral tegmental area (VTA) projection induced depression‐like behaviors in TN mice [23]. As a highly heterogeneous brain area, PBN comprises diverse neuronal subtypes, each implicated in distinct physiological functions [24]. For instance, *Tacr1*‐expressing neurons localized in the dorsal subarea are specifically involved in processing nociceptive signals originating from extracranial regions [25], while neighboring cholecystokinin (*Cck*)‐expressing neurons play a critical role in thermoregulation [26]. Conversely, external lateral PBN (PBel) located neuronal populations, including Calcitonin Gene‐Related Peptide (*Calca*)‐expressing neurons [27, 28, 29], *Tac1*‐expressing neurons [30], and opioid receptor μ1 (*Oprm1*)‐expressing neurons [31], have been implicated in fear processing, pain modulation, and respiratory control, respectively. Despite these advances, the specific neuronal identities and underlying mechanisms by which PBN modulates TN‐induced pain hypersensitivity remain poorly defined.

In the present study, we employed the CION‐induced TN mouse model [32, 33] combined with a head‐fixation paradigm to systematically evaluate both spontaneous and stimulus‐evoked pain‐related behaviors. Our findings uncovered a critical TG‐Sp5C^Tac1^‐PBN^Tac1^ neural circuit that plays an essential role in mediating TN‐induced pain hypersensitivity.

## 2 Results

### 2.1 PBN^Tac1^ Neurons Critically Modulate TN‐Induced Pain Hypersensitivity

**FIGURE 1:**
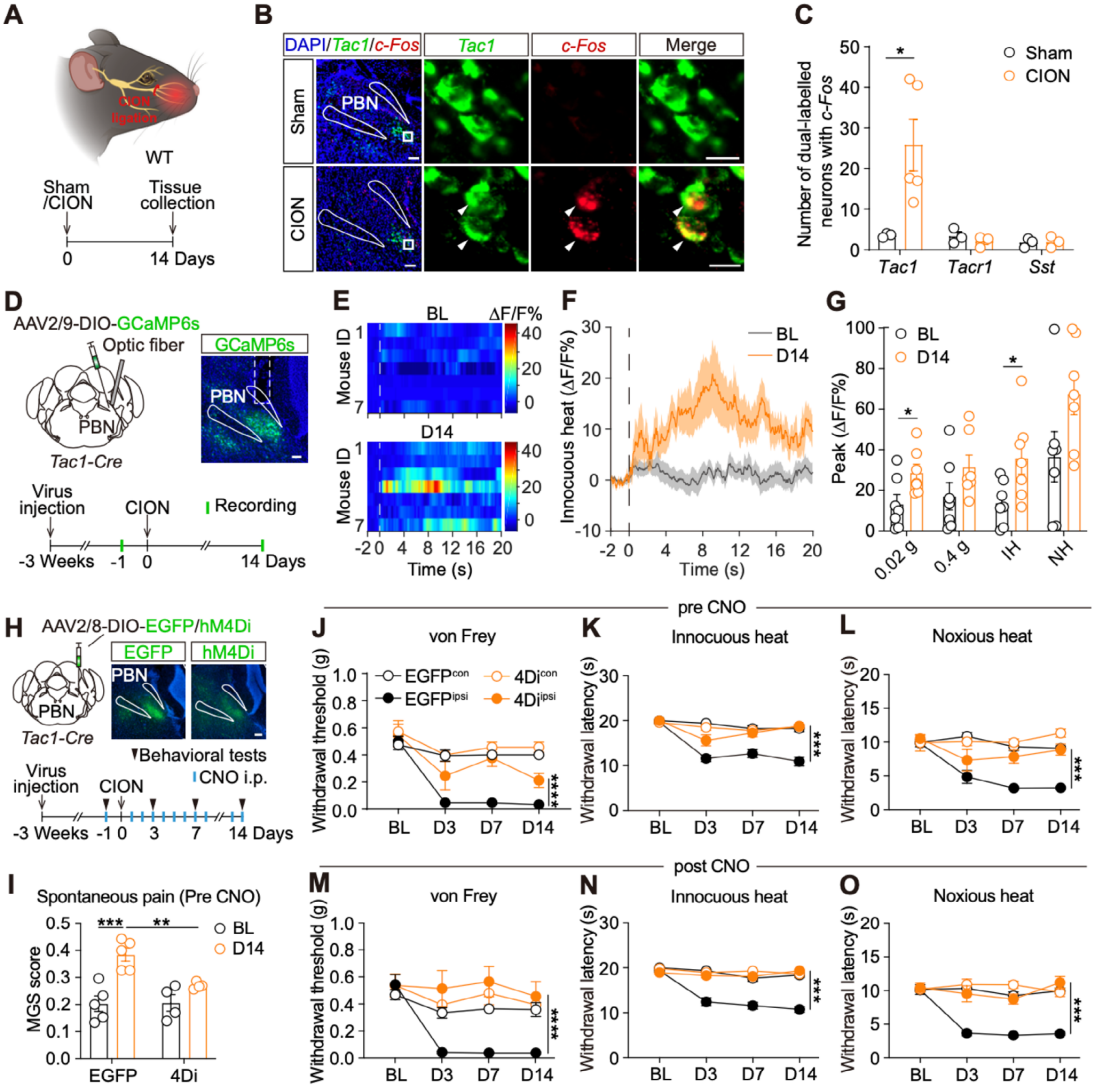
PBN^Tac1^ neurons critically modulate TN‐induced pain hypersensitivity. (A), A schematic for sham/CION surgery, and a timeline for brain tissue collection. (B), The images showing examples of gene expression pattern of *c‐Fos* (red) and *Tac1* (green) in the right PBN from mice in both groups. Blue: DAPI. Scale bar: 100 µm (left), 20 µm (right). (C), Average number of *c‐Fos* co‐labeled neurons in distinct cell types expressed in the right PBN from mice in both groups. ^*^ *p* < 0.05, by Mann Whitney test. (D), A schematic and timeline for fiber photometry recording of PBN^Tac1^ neurons. Images showing an example of GCaMP6s virus expression pattern and optical fiber implantation site in PBN. Scale bar: 100 µm. (E), Heatmaps showing innocuous thermal stimulus‐evoked fluorescence changes recorded at baseline (BL) and day 14 after CION surgery (D14). (F), Curves showing average innocuous heat stimulus‐evoked fluorescence signals. Dash line represents the onset of stimuli. (G), Peak value of distinct stimulus‐evoked fluorescence responses recorded from PBN^Tac1^ neurons (IH: innocuous heat; NH: noxious heat, dots represent the average peak of each mouse). ^*^ *p* < 0.05, by paired t test (n = 7 mice in each group). (H), Schematic and timeline for the experiment. The example images showing EGFP and hM4Di virus expression patterns in PBN. Scale bar: 100 µm. (I), MGS score measured from mice in both groups. ^***^ *p* < 0.001, by two‐way ANOVA (EGFP, n = 5; hM4Di, n = 4). (J–O), Mechanical withdrawal responses evoked by von Frey filaments (J, M), and thermal withdrawal latency evoked by innocuous heat (K, N) and noxious heat (L, O) measured in mice from both groups before and after the application of CNO. ^*^ *p* < 0.05, ^***^ *p* < 0.001, by two‐way ANOVA (EGFP, n = 8; hM4Di, n = 7).

To systematically and efficiently measure the orofacial mechanical/thermal pain threshold and spontaneous pain behaviors in mice, we employed a customized head‐fixation system (Figure S1A). After the habituation to the head‐fixation system for 3 consecutive days, spontaneous pain behaviors, von Frey filament‐evoked mechanical responses (withdrawal threshold), and infrared ray‐evoked withdrawal responses (withdrawal latency) to both innocuous and noxious thermal stimuli were measured in the vibrissal pad (Figure S1B). Spontaneous pain was evaluated using the Mouse Grimace Scale (MGS) [34, 35]. For each mouse, 60 frames were sampled from a 10‐min video recording (Figure S1C). The MGS score for each frame was calculated by averaging the scores across five distinct facial regions, and the final MGS score for each mouse was defined as the average MGS derived from 60 frames (Figure S1D). We measured pain‐related behaviors in mice from both CION and sham groups (Figure 1A). Compared with the sham controls, the CION group exhibited a significantly higher MGS score on day 14 post‐surgery (Figure S1D,E), together with a decreased mechanical withdrawal threshold (Figure S1F) and a shorter thermal withdrawal latency in the ipsilateral vibrissal pad starting from day 3 post‐surgery (Figure S1G,H). These results are consistent with previous studies [32, 36], confirming the validity of our behavioral testing paradigms and the successful establishment of the CION‐induced TN model.

PBN has been reported to receive both direct and indirect inputs from the TG [2, 6, 19, 20, 21] and exhibits increased activity in TN models [37]. To investigate the role of the PBN in modulating TN‐induced pain hypersensitivity, we injected the AAV2/8‐hM4Di virus (encoding the inhibitory DREADD receptor hM4Di) into the right PBN of wild‐type (WT) mice to chemogenetically inhibit the right PBN neurons, with AAV2/8‐EGFP virus as a control (Figure S1I,L). After three weeks of virus expression, we assessed the baseline withdrawal threshold and observed no significant differences between the PBN inhibition (hM4Di) and control (EGFP) groups (Figure S1J,K). Subsequently, we performed CION surgery and intraperitoneally (i.p.) administrated clozapine N‐oxide (CNO) daily for two weeks to continuously inhibit PBN neurons. The mechanical thresholds of all mice were serially tested post‐surgery. We found that chemogenetic inhibition of the right PBN significantly prevented the TN‐induced mechanical pain hypersensitivity (Figure S1I–K). More importantly, mice in the hM4Di group showed attenuated pain hypersensitivity starting from day 7, even prior to CNO administration, suggesting the activity of PBN neurons is critical for the initiation of TN‐induced pain hypersensitivity (Figure S1J,K).

The PBN is a heterogeneous nucleus [38]. To further define the neuronal subtypes involved in TN modulation, we examined the co‐localization of *c‐Fos* with molecular markers expressed in PBN, including somatostatin (*Sst*), *Tacr1*, and *Tac1*, using RNAscope in situ hybridization (ISH) in both sham and CION groups (Figure 1A–C; Figure S1M,N). Since most *c‐Fos‐*positive neurons were localized in the PBel, we quantified neurons in this subarea and observed that the average number of *c‐Fos* co‐localized *Tac1*‐positive neurons was significantly higher in the CION group compared with the sham group (25.8 vs 3.6 cells) (Figure 1C). This finding suggests that a greater number of PBN^Tac1^ neurons were activated in the TN model. To further investigate the activity of PBN^Tac1^ neurons, we utilized fiber photometry during the development of TN in vivo. After the injection of AAV2/9‐DIO‐GCaMP6s virus and the implantation of optical fibers into the right PBN for three weeks, we measured the orofacial stimulus‐evoked fluorescence signals before and after the CION surgery (Figure 1D; Figure S2A,B). Consistent with the *c‐Fos* staining results, we observed a significantly increase in stimulus‐evoked responses of PBN^Tac1^ neurons on day 14 post‐surgery compared with baseline levels (Figure 1E–G). Overall, our data revealed PBN^Tac1^ neurons exhibit increased activity in the CION model, suggesting their involvement in TN modulation.

To further define the functional role of PBN^Tac1^ neurons in TN modulation, we employed a chemogenetic approach. Specifically, AAV2/8‐DIO‐hM4Di‐EGFP virus was injected into the right PBN of *Tac1*‐*Cre* mice, with the AAV2/8‐DIO‐EGFP virus as a control. Following a three‐week period for virus expression, right‐sided CION surgery was performed. Subsequently, CNO was administered (i.p.) daily for two weeks (Figure 1H). We found that chronic inhibition of PBN^Tac1^ neurons significantly reduced the TN‐induced pain hypersensitivity. This was evidenced by a lower MGS score, an increased mechanical withdrawal threshold, and a prolonged thermal withdrawal latency in the hM4Di group compared with the control group (Figure 1I–O). These findings highlight the critical role of right PBN^Tac1^ neurons in modulating TN. More importantly, similar to the finding from inhibition of all neurons in PBN (Figure S1J), we observed an enhanced pain threshold before CNO administration, beginning from day 7 (six days after CNO application) (Figure 1J–L). These data suggest TN‐induced neuroplasticity is reversed following chronic inhibition of PBN^Tac1^ neurons.

A previous study reported that activation of PBN^Tac1^ neurons increases breathing rate [39]. To further define whether the analgesic effect we observed following inhibition of PBN^Tac1^ neurons is due to altered breathing states, we employed the plethysmographic measurements. We found that the inhibition of PBN^Tac1^ neurons had no effect on breathing rate (Figure S2C–H). These data suggest the analgesic effect is not induced by the alteration of breathing state.

*Oprm1*‐expressing PBN (PBN^Oprm1^) neurons represent another PBel‐located cell type and contribute to affective pain [31]. To further define the role of PBN^Oprm1^ neurons in TN modulation, we employed the same virus injection and behavioral test paradigm (Figure S2I,M). Our results revealed that inhibition of PBN^Oprm1^ neurons did not significantly alleviate TN‐induced pain hypersensitivity (Figure S2J–L). These findings highlight the specific role of PBN^Tac1^ neurons in TN modulation.

### 2.2 PBN‐Projecting Sp5C Neurons are Essential for TN Pathology

**FIGURE 2:**
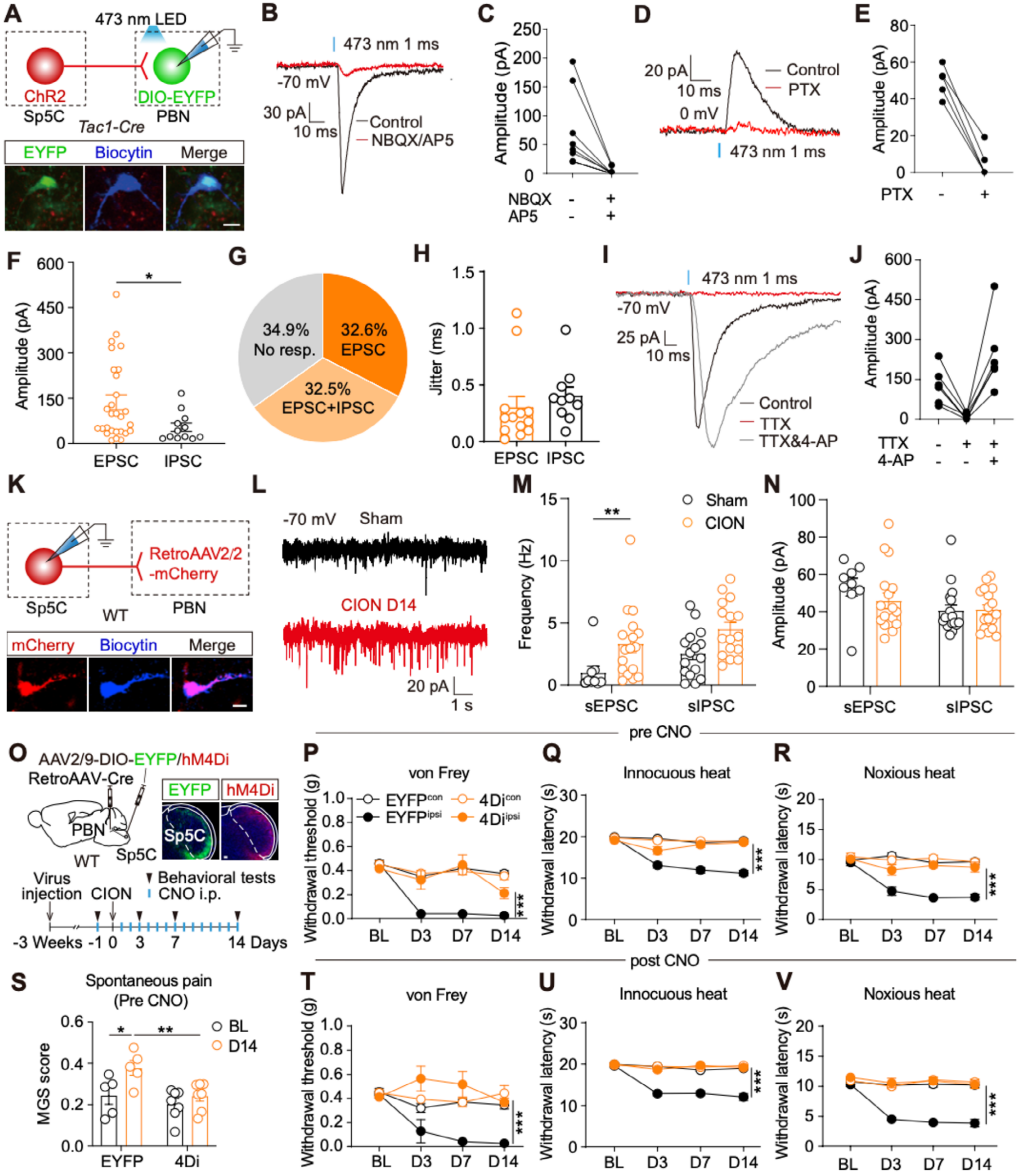
PBN‐projecting Sp5C neurons are essential for TN development. (A), A schematic for electrophysiological recording. Images showing virus expression and a biocytin‐filled recorded neuron in PBN. Blue: biocytin; Red: ChR2‐positive fiber from Sp5C; Green: EYFP‐positive PBN^Tac1^ neuron. Scale bar: 10 µm. (B), Examples of light‐evoked inward currents recorded from PBN^Tac1^ neurons at baseline (black), after application of NBQX/AP5 (red). (C), Amplitudes of light‐evoked EPSCs recorded without and with NBQX/AP5 (n = 8 neurons from 2 mice). (D), Examples of light‐evoked outward currents recorded from PBN^Tac1^ neurons at baseline (black), after application of picrotoxin (red). (E), Amplitudes of light‐evoked IPSCs recorded without and with picrotoxin (n = 5 neurons from 3 mice). (F), Amplitudes of light‐evoked EPSCs and IPSCs. ^*^ *p* < 0.05, by Mann Whitney test (n = 28 neurons for EPSC, and n = 12 neurons for IPSC from 11 mice). (G), Proportion of neurons with different response patterns (n = 43 neurons from 11 mice). (H) Jitter analysis of EPSC and IPSC detected in PBN‐projecting Sp5C neurons (EPSC, n = 13 neurons from 5 mice; IPSC, n = 10 neurons from 3 mice). (I), Examples of light‐evoked EPSCs recorded from PBN^Tac1^ neurons at baseline (black), after application of TTX (red), and further addition of 4‐AP (gray). (J), Amplitudes of light‐evoked EPSCs recorded under distinct conditions (n = 6 neurons from 4 mice). (K), A schematic for electrophysiological recording. Example images showing one recorded tdTomato‐positive PBN‐projecting Sp5C neuron (red). Blue: biocytin. Scale bar: 10 µm. (L), Examples showing the sEPSC recorded from neurons in both groups. (M), Frequencies of sEPSC/sIPSC inputs to PBN‐projecting Sp5C^Tac1^ neurons in both groups. ^**^ *p* < 0.001, by Mann Whitney test ^*^ *p* < 0.05, by unpaired t test (sham: n = 9 neurons for sEPSC, and n = 17 neurons for sIPSC from 2 mice; CION: n = 17 neurons for sEPSC, and n = 16 neurons for sIPSC from 2 mice). (N), Amplitudes of sEPSC/sIPSC of PBN‐projecting Sp5C^Tac1^ neurons in both groups. (sham: n = 9 neurons for sEPSC, and n = 17 neurons for sIPSC from 2 mice; CION: n = 17 neurons from 2 mice). (O), A schematic and timeline for the experiment. Images showing examples of EYFP and hM4Di virus expression patterns in PBN. Scale bar: 100 µm. (P–V), MGS score (S, EYFP, n = 5; hM4Di, n = 7), withdrawal threshold (P, T), withdrawal latency of heat allodynia (Q, U), and heat hyperalgesia (R, V) measured in mice from both groups before and after the application of CNO. ^*^ *p* < 0.05, ^**^ *p* < 0.01, ^***^ *p* < 0.001, by two‐way ANOVA (EYFP, n = 10; hM4Di, n = 12).

To map the upstream circuits of the PBN, we injected CTB‐555 into the PBN to retrogradely label its upstream neurons (Figure S3A). One week after the injection, CTB‐555‐positive neurons were detected in the Sp5C and the NTS (Figure S3B). Given that the Sp5C has been reported to be involved in TN modulation [40], we further investigated the Sp5C to PBN pathway by injecting AAV2/8‐EGFP.PreSynapse virus into the right Sp5C of WT mice (Figure S3C). After 3 weeks of virus expression, EGFP‐positive axon terminals were detected in both the right PBN and the right ventral posteromedial nucleus of the thalamus (VPM) (Figure S3D). These tracing results indicate Sp5C is one of the upstream regions of the PBN. To further define the functional connectivity between Sp5C and PBN^Tac1^ neurons, we utilized the electrophysiological method. AAV2/9‐ChR2‐mCherry virus was injected into the right Sp5C to express ChR2 in Sp5C neurons, and AAV2/9‐DIO‐EYFP virus was injected into the right PBN of *Tac1*‐*Cre* mice to label PBN^Tac1^ neurons (Figure 2A). After virus expression for 3 weeks, we recorded the blue light‐evoked responses in EYFP‐labeled PBN^Tac1^ neurons and detected inward currents (Figure 2B). These inward currents were blocked by the bath application of NBQX/AP5, the antagonist of AMPA/NMDA receptors, suggesting they are excitatory postsynaptic currents (EPSCs) (Figure 2B,C). In some PBN^Tac1^ neurons that receive EPSCs input, we also detected outward currents (Figure 2D). These outward currents were blocked by bath application of picrotoxin, the antagonist of GABA receptors, suggesting they are inhibitory postsynaptic currents (IPSCs) (Figure 2D,E). In consideration of the connectivity and amplitude of the inhibitory connections, which were significantly lower than those of the excitatory connections (Figure 2F,G), these findings suggest Sp5C neurons primarily send excitatory projections to PBN^Tac1^ neurons. The jitter of both EPSCs and IPSCs was predominantly under 1 ms, indicating monosynaptic connections (Figure 2H). The monosynaptic nature of the excitatory connections was further confirmed by the observation that the EPSCs were blocked by tetrodotoxin (TTX) and subsequently reversed by the further addition of 4‐aminopyridine (4‐AP) (Figure 2I,J). Together, these results suggest Sp5C neurons primarily send monosynaptic excitatory projections to PBN^Tac1^ neurons.

To investigate the role of Sp5C in TN modulation in vivo, we injected AAV2/8‐hM4Di‐mCherry virus into the right Sp5C to chemogenetically inhibit neurons in this region, with AAV2/8‐EGFP virus as a control (Figure S3E,H). Three weeks after the virus injection, we performed the CION surgery and monitored the mechanical threshold of the vibrissal pad following daily CNO administration (Figure S3E). We observed that chronic inhibition of Sp5C neurons significantly alleviated the CION‐induced pain hypersensitivity (Figure S3F,G), suggesting a crucial role of Sp5C neurons in TN modulation. Among all Sp5C neurons, only a subpopulation of neurons located in the superficial layers project to PBN (Figure S3A,B). To further explore whether these PBN‐projecting Sp5C neurons contribute to TN modulation, we first defined the TN‐induced neuroplasticity of PBN‐projecting Sp5C neurons. This was achieved by injecting RetroAAV2/2‐mCherry virus into PBN to label PBN‐projecting Sp5C neurons, followed by recording mCherry‐positive PBN‐projecting Sp5C neurons 14 days after surgery in both sham and CION groups (Figure 2K). We observed that the frequency of both spontaneous excitatory postsynaptic current (sEPSC) and spontaneous inhibitory postsynaptic current (sIPSC) recorded from the CION group was significantly higher than that from the sham group, while the amplitude of sEPSC or sIPSC remained comparable between two groups (Figure 2L–N). The increased excitatory and inhibitory inputs to PBN‐projecting Sp5C neurons in the CION model suggest that TN induces higher complicated presynaptic effect on PBN‐projecting Sp5C neurons.

To further define the plasticity of the Sp5C‐PBN pathway in the CION model, we injected AAV2/9‐GCaMP6s virus into the right Sp5C, implanted optical fibers into the right PBN, and recorded the stimulus‐evoked responses of the Sp5C‐PBN pathway both pre‐ and 14 days post‐surgery (Figure S4A,B). We observed significantly increased stimulus‐evoked responses in the CION group on day 14 compared with baseline (Figure S4F–H), while this phenomenon was not observed in the sham group (Figure S4C–E). Our data revealed that the activity of the Sp5C‐PBN pathway increased in the TN model, suggesting the crucial role of PBN‐projecting Sp5C neurons in TN modulation. To test this hypothesis, we injected RetroAAV2/2‐Cre virus into the right PBN and AAV2/9‐DIO‐hM4Di‐mCherry virus into the right Sp5C to specifically express hM4Di in PBN‐projecting Sp5C neurons of WT mice, with AAV2/9‐DIO‐EYFP injected into Sp5C as a control (Figure 2O; Figure S4I). After virus expressing for 3 weeks, we monitored the pain threshold of mice in both groups before and after CION surgery with daily CNO administration (Figure 2O). We observed that chronic inhibition of PBN‐projecting Sp5C neurons significantly reduced CION‐induced pain hypersensitivity (Figure 2P–V). Moreover, we noticed that pain relief occurred on day 3 prior to the administration of CNO (Figure 2P–R), indicating the inhibition of PBN‐projecting Sp5C neurons for two days is sufficient to block the development of TN‐induced pain hypersensitivity.

To investigate whether activating the Sp5C‐PBN pathway could induce spontaneous orofacial pain‐related wiping behaviors, we employed optogenetic techniques. Specifically, we injected RetroAAV2/2‐Cre virus into the right PBN and AAV2/9‐DIO‐ChR2‐EYFP virus into the right Sp5C of WT mice, with AAV2/9‐DIO‐EYFP injected into Sp5C as a control. Optical fibers were then implanted into the PBN to activate the Sp5C‐PBN pathway (Figure S4J). After allowing virus expression for three weeks, we conducted behavioral tests and observed a significant increase in light‐evoked spontaneous wiping behaviors in the ChR2 group compared with the control group (Figure S4K–M). We also assessed the pain threshold during both light‐on and light‐off periods. In the ChR2 group, we observed a significant decrease in mechanical withdrawal threshold during the light‐on period compared with that of the light‐off period, whereas no such difference was observed in the EYFP group (Figure S4N,O). These results suggest Sp5C‐PBN pathway plays a significant role in orofacial pain processing. Given that pain sensation is often associated with aversion [41], we conducted a real‐time place avoidance (RTPA) test. We observed that mice in the ChR2 group spent less time in the light‐stimulating chamber during the light‐stimulation period compared with the pre‐stimulation phase, although this difference did not reach statistical significance (Figure S4P,Q). Our data demonstrate that activation of the Sp5C‐PBN pathway is sufficient to induce spontaneous pain‐related wiping behaviors and pain hypersensitivity in the vibrissal pad.

Taken together, we found that Sp5C sends projections to PBN, and PBN‐projecting Sp5C neurons play both necessary and sufficient roles in TN modulation.

### 2.3 PBN‐Projecting Sp5C Neurons are Primarily *Tac1*‐Positive

**FIGURE 3:**
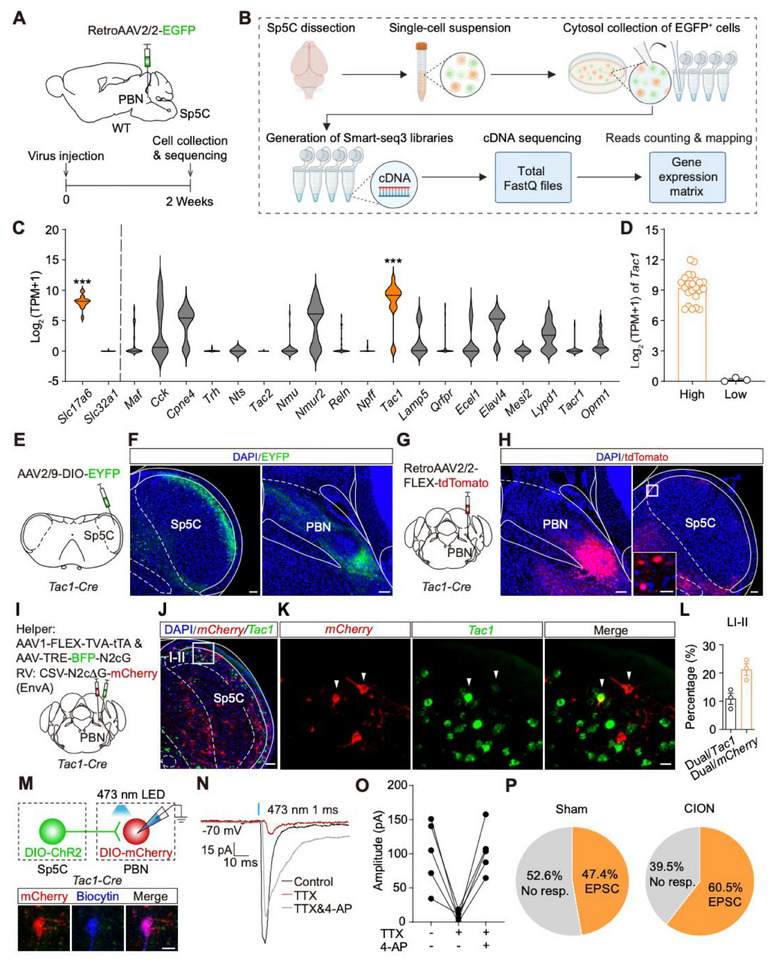
Sp5C^Tac1^ neurons have monosynaptic connections with PBN^Tac1^ neurons. (A), A schematic and timeline for the single‐neuron sequencing experiment. (B), A schematic for the procedure of single‐cell sequencing of PBN‐projecting Sp5C neurons. (C), The expression levels of inhibitory and excitatory marker genes and molecular marker genes in PBN‐projecting Sp5C neurons. The top expressed genes *Slc17a6* and *Tac1* in these two groups were highlighted in orange. ^***^ *p* < 0.001, by Mann Whitney test (n = 26 neurons from 3 mice). (D), Distribution of *Tac1* gene expression level in neurons we sampled (n = 26 neurons from 3 mice). (E), A schematic for anterograde tracing. (F), Images showing the distribution of EYFP‐labeled Sp5C^Tac1^ neurons (left) and their axons in PBN (right). Green: EYFP, blue: DAPI. Scale bar: 100 µm. (G), A schematic for retrograde tracing. (H), Images showing the expression pattern of the virus in PBN (left) and their upstream *Tac1*‐positive neurons in Sp5C (right). Red: tdTomato, blue: DAPI. Scale bar: 100 µm. Inset scale bar: 20 µm. (I), A schematic for rabies virus‐based retrograde tracing. (J), Distribution pattern of *mCherry*‐labeled RV‐positive neurons (red) and *Tac1*‐positive (green) neurons in the right Sp5C. Blue: DAPI. Scale bar: 100 µm. (K), Example images showing *mCherry*‐labeled RV‐positive neurons (red) and *Tac1*‐positive neurons (green) in the superficial layers of the right Sp5C. Arrows pointing to the dual‐labeled neurons. Scale bar: 20 µm. (L), Percentage of dual‐labeled neurons in *Tac1* or RV‐positive neurons measured in the laminae I‐II of the right Sp5C. (M), A schematic for electrophysiological recording. Example images showing one recorded mCherry‐positive PBN^Tac1^ neuron. Red: PBN^Tac1^ neuron; Blue: biocytin. Scale bar: 10 µm. (N), Example traces of light‐evoked EPSCs recorded from PBN^Tac1^ neurons at baseline (black), after application of TTX (red), and further addition of 4‐AP (gray). (O), Amplitudes of light‐evoked EPSCs recorded under distinct conditions (n = 5 neurons from 2 mice). (P), Proportion of neurons recorded from mice in both groups that showed light‐evoked responses or no responses (sham: n = 19 neurons from 4 mice; CION: n = 38 neurons from 3 mice).

To identify the molecular makers labeling PBN‐projecting Sp5C neurons, we conducted single‐neuron sequencing on these neurons. We injected RetroAAV2/2‐EGFP virus into the right PBN of WT mice to express EGFP in PBN‐projecting Sp5C neurons (Figure 3A). After two weeks of virus expression, we collected the superficial tissue of Sp5C and digested it into a single‐cell suspension, then manually selected EGFP‐labeled neurons under the microscope. Subsequently, we constructed a Smart‐seq3 library for each neuron and performed the following sequencing and data analysis (Figure 3B). We calculated and compared the log_2_ (TPM+1) values of excitatory and inhibitory marker genes, and found that PBN‐projecting Sp5C neurons predominantly express excitatory neuronal marker *Slc17a6* (Figure 3C). This finding is consistent with our electrophysiological recording data (Figure 2K–M), indicating PBN‐projecting Sp5C neurons are excitatory in nature. We next analyzed the expression levels of genes previously reported to be expressed in excitatory neurons and spinal projection neurons [11], including *Cck*, Copine IV (*Cpne4*), Neuromedin U receptor 2 (*Nmur2*)*, Oprm1*, and *Tac1*, among others, in PBN‐projecting Sp5C neurons (Figure 3C). Among all genes we analyzed, the expression level of *Tac1* gene was significantly higher than other marker genes (Figure 3C). Additionally, *Tac1* was robustly expressed in 23 (Log_2_ (TPM+1) > 6) out of 26 neurons (Figure 3D). These findings strongly suggest that the *Tac1* gene serves as a specific marker for PBN‐projecting Sp5C neurons. To further validate the sequencing data, we characterized the projection pattern of distinct types of neurons located in the superficial layers of the Sp5C, including *Tac1*‐, *Tacr1*‐, *Pdyn*‐ and *Nmur2*‐positive neurons, according to the identification of spinal projection neurons [12] and the superficial location properties of projection neurons. Specifically, we injected AAV2/9‐fDIO‐hM4Di‐mCherry virus into the right Sp5C of *Tacr1‐FLPo* mice, or AAV2/9‐DIO‐EYFP virus into the right Sp5C of *Tac1‐Cre*, *Pdyn*‐*Cre*, and *Nmur2*‐*Cre* mice (Figure 3E; Figure S5A). Three weeks after virus expression, EYFP‐positive fibers were detected in the right PBN of *Tac1*‐*Cre* mice (Figure 3F; Figure S5C). In contrast, few mCherry/EYFP fibers were detected in the PBN of *Tacr1‐FLPo*, *Pdyn*‐*Cre* or *Nmur2*‐*Cre* mice (Figure S5B). These results further confirmed that *Tac1*‐positive Sp5C (Sp5C^Tac1^) neurons, rather than other cell types we tested, are able to project to the PBN. Additionally, we injected RetroAAV2/2‐FLEX‐tdTomato virus into the PBN of *Tac1*‐*Cre* mice to label PBN‐projecting Sp5C^Tac1^ neurons (Figure 3G). Three weeks after the injection, we observed tdTomato‐positive neurons presented in Sp5C (Figure 3H; Figure S5D), indicating the PBN indeed receives projection from Sp5C^Tac1^ neurons. Collectively, our sequencing and tracing data suggest that PBN‐projecting Sp5C neurons are mainly *Tac1‐*positive.

To further determine whether Sp5C^Tac1^ neurons directly project to PBN^Tac1^ neurons, we employed retrograde tracing experiments using rabies virus (RV). Briefly, we injected the helper virus, AAV1‐FLEX‐split TVA‐P2A‐EGFP‐P2A‐tTA and AAV‐TREtight‐mTagBFP2‐N2cG, into the right PBN of *Tac1*‐*Cre* mice to express G proteins in the PBN^Tac1^ neurons. One week after the helper virus injection, we injected the RV virus CSV‐N2ΔG‐4mCherry (EnvA) in the right PBN to label PBN^Tac1^ neurons and their presynaptic neurons in the brain (Figure 3I; Figure S5E,F). One week following the RV injection, RV‐positive neurons were observed in multiple upstream areas across both the ipsilateral and contralateral hemispheres, including the Sp5C, the central amygdala nucleus (CEA), and the somatosensory areas (SS) (Figure S5G–I). To confirm whether RV‐positive neurons in Sp5C are *Tac1‐*positive, we performed RNAscope ISH on Sp5C sections, and found that 21.3% of RV‐positive neurons located in laminae I‐II of the Sp5C are *Tac1‐*positive (Figure 3J–L). These data indicate PBN^Tac1^ neurons receive a direct projection from Sp5C^Tac1^ neurons. To define the functional connections between these two populations, we utilized electrophysiological recording combined with the injection of AAV2/9‐DIO‐ChR2‐EYFP virus into Sp5C of *Tac1‐Cre* mice to express ChR2 in Sp5C^Tac1^ neurons, and AAV2/9‐DIO‐mCherry virus into PBN to label PBN^Tac1^ neurons with mCherry (Figure 3M). After the virus expression for 3 weeks, we prepared the PBN slices and recorded light‐evoked responses in PBN^Tac1^ neurons. We detected blue light‐evoked EPSC in PBN^Tac1^ neurons, which were blocked by the application of TTX and subsequently reversed following the further application of 4‐AP, indicating a monosynaptic connection between Sp5C^Tac1^ neurons and PBN^Tac1^ neurons (Figure 3N,O). Different from both excitatory and inhibitory connections between Sp5C neurons and PBN^Tac1^ neurons (Figure 2B–E), our results showed that only excitatory connections were detected between Sp5C^Tac1^ neurons and PBN^Tac1^ neurons (Figure 3P). To further determine whether the connection between Sp5C^Tac1^ and PBN^Tac1^ neurons is altered in the TN model, we recorded this connection from mice in both the sham and CION groups. Our analysis revealed that the connectivity between Sp5C^Tac1^ neurons and PBN^Tac1^ neurons was significantly increased in mice from the CION group compared to the sham group (Figure 3P). This enhanced connectivity indicates the involvement of this Sp5C^Tac1^‐PBN^Tac1^ pathway in TN modulation.

### 2.4 The Sp5C^Tac1^‐PBN Pathway Plays Crucial Roles in TN Modulation

To investigate the role of Sp5C^Tac1^ neurons in TN modulation, we examined the co‐localization of *Tac1* and *c‐Fos* genes in Sp5C from mice in the sham and CION groups. Our analysis revealed that in the superficial layers of Sp5C, the proportion of *c‐Fos‐*positive neurons within *Tac1‐*positive neurons was significantly higher in the CION group compared to the sham control (Figure S6A,B). This finding suggests that a greater number of Sp5C^Tac1^ neurons were activated in the TN model. To further test the functional role of Sp5C^Tac1^ neurons in TN modulation, we injected AAV2/9‐DIO‐hM4Di‐mCherry virus into Sp5C of the *Tac1‐Cre* mice, with AAV2/9‐DIO‐EYFP virus as a control. After the virus expression, we did CION surgery with mice in both groups, injected CNO daily and performed behavioral tests (Figure S6C,D,K). We found that the inhibition of Sp5C^Tac1^ neurons significantly reduced TN‐induced pain hypersensitivity (Figure S6H–J). However, before the CNO application, we observed no significant changes in pain hypersensitivity changes in hM4Di group compared with the EYFP group, suggesting that chronic inhibition of Sp5C^Tac1^ neurons does not exert a persistent effect on pain relief (Figure S6E–G). This lack of persistent effect may be attributed to the heterogeneity of Sp5C^Tac1^ neurons.

**FIGURE 4:**
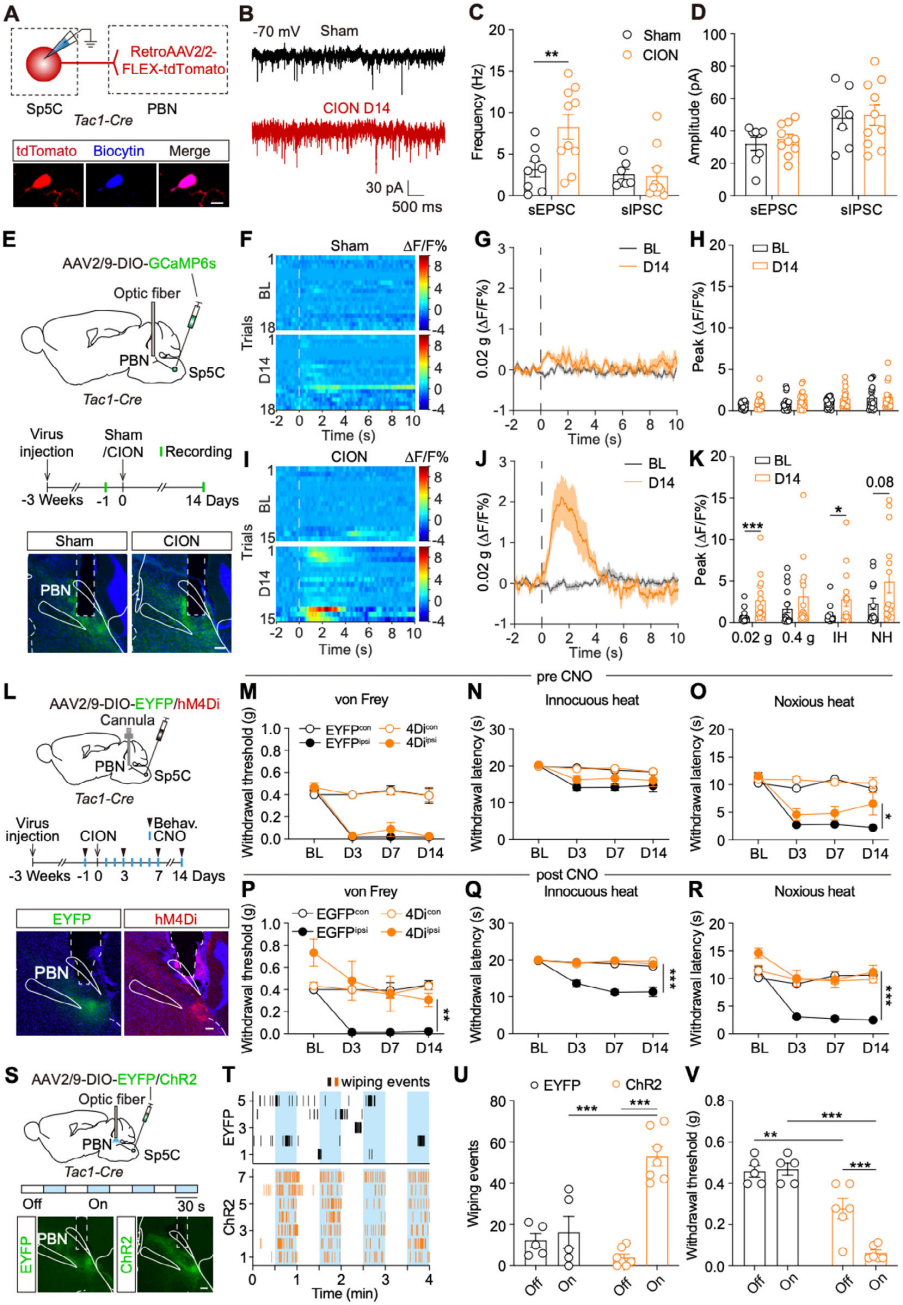
Sp5C^Tac1^‐PBN circuit contributes to TN. (A), A schematic for electro‐physiological recording. Example images showing one recorded tdTomato‐labeled PBN‐projecting Sp5C^Tac1^ neuron. Red: PBN‐projecting Sp5C^Tac1^ neuron; Blue: biocytin. Scale bar: 10 µm. (B), Examples showing the spontaneous EPSCs recorded at ‐70 mV from PBN‐projecting Sp5C^Tac1^ neuron in both groups. (C), Frequencies of spontaneous EPSCs/IPSCs recorded from PBN‐projecting Sp5C^Tac1^ neurons in both groups. ^*^ *p* < 0.05, by unpaired t test. (sham: n = 8 neurons for sEPSC, and n = 7 neurons for sIPSC from 3 mice; CION: n = 10 neurons from 4 mice). (D), Amplitudes of spontaneous EPSCs/IPSCs recorded from PBN‐projecting Sp5C^Tac1^ neurons in both groups (sham: n = 8 neurons for sEPSC, and n = 7 neurons for sIPSC from 3 mice; CION: n = 10 neurons from 4 mice). (E), A schematic and timeline for fiber photometry recording of the right Sp5C^Tac1^‐PBN pathway. Images showing examples of virus expression patterns and optical fiber implantation sites in the PBN. Scale bar: 100 µm. (F–K), Heatmaps showing innocuous 0.02 g von Frey filament‐evoked fluorescence changes recorded from mice in the sham (F) and CION (I) groups. Curves showing 0.02 g von Frey filament‐evoked fluorescence signals recorded from mice in the sham (G) and CION (J) groups. Dash line represents the onset of stimuli. Peak values of stimulus‐evoked fluorescence signals recorded from mice in sham (H) and CION (K) groups. BL: baseline; D14: day 14 after CION surgery. ^*^ *p* < 0.05, ^***^ *p* < 0.001 by Mann Whitney test (Sham, n = 18 trials from 6 mice; CION, n = 15 trials from 5 mice). (L), A schematic for chemogenetic inhibition of the Sp5C^Tac1^‐PBN pathway. The images showing examples of the distribution pattern of EYFP‐ and hM4Di‐ positive axons, and the location of cannula (dash line) in the PBN. Scale bar: 100 µm. (M–R), Mechanical withdrawal threshold (M, P), innocuous heat (N, Q) noxious heat (O, R) evoked withdrawal latency measured in mice from both groups before and after the application of CNO. ^***^ *p* < 0.001, by two‐way ANOVA (EYFP, n = 5; hM4Di, n = 6). (S), A schematic for optogenetic activation of the Sp5C^Tac1^ ‐ PBN pathway. The images showing examples of EYFP and ChR2 virus expression patterns and optical fiber implantation sites in the PBN. Scale bar: 100 µm. (T), Raster plots of wiping behavior observed in mice from EYFP and ChR2 groups. Blue rectangles represent blue laser‐on period (EYFP, n = 5; ChR2, n = 7). (U), Wiping events observed in mice from two groups during laser‐off and laser‐on. ^***^ *p* < 0.001, by two‐way ANOVA. (V), Mechanical threshold of the right vibrissal pad was detected from mice in both groups during laser‐on and laser‐off period. ^***^ *p* < 0.001, by two‐way ANOVA (EYFP, n = 5; ChR2, n=6 mice).

To determine if PBN‐projecting Sp5C^Tac1^ neurons show TN‐induced plasticity, we utilized electrophysiological recordings. RetroAAV2/2‐FLEX‐tdTomato virus was injected into the PBN of *Tac1‐Cre* mice to label PBN‐projecting Sp5C^Tac1^ neurons (Figure 4A), followed by recording tdTomato‐labeled neurons on day 14 after the CION or sham surgery. Compared with the sham group, PBN‐projecting Sp5C^Tac1^ neurons in the CION group exhibited a significant increase in frequency of sEPSC, but not sIPSC (Figure 4B,C). However, the amplitudes of neither sEPSC nor sIPSC had any significant difference between the sham and CION groups (Figure 4D). These data suggest TN specifically enhances excitatory inputs to PBN‐projecting Sp5C^Tac1^ neurons.

To evaluate the plasticity of the Sp5C^Tac1^‐PBN pathway in vivo, we injected AAV2/9‐DIO‐GCaMP6s virus into the right Sp5C, and implanted optical fibers into the right PBN of *Tac1*‐*Cre* mice. Three weeks after virus expression, baseline stimulus‐evoked activity of this pathway was recorded prior to CION/sham surgery, with repeated recording on day 14 post‐surgery (Figure 4E; Figure S6L). We found that stimulus‐evoked responses were significantly increased on day 14 in the CION group compared with the baseline, which was not observed in the sham group (Figure 4F–K). Collectively, our ex vivo electrophysiological recording and in vivo fiber photometry recording data demonstrate that TN enhances both excitatory inputs to Sp5C^Tac1^ neurons and the activity of the Sp5C^Tac1^‐PBN pathway, indicating the involvement of this pathway in TN modulation.

To investigate the role of Sp5C^Tac1^‐PBN pathway in TN modulation, we injected AAV2/9‐DIO‐hM4Di‐EYFP virus into Sp5C of *Tac1*‐*Cre* mice, with AAV2/9‐DIO‐EYFP virus as a control. The intracranial cannula was implanted into PBN to specifically inhibit the Sp5C^Tac1^‐PBN pathway with daily CNO infusion (Figure 4L; Figure S6M). Behaviors of mice in both groups were assessed before and after CNO administration on distinct days. We found that inhibiting the Sp5C^Tac1^‐PBN pathway significantly alleviated the TN‐induced pain hypersensitivity (Figure 4P–R). While no analgesic effect was observed prior to CNO application (Figure 4M–O), suggesting axonal inhibition is not sufficient to reverse the TN‐induced plasticity of PBN‐projecting Sp5C^Tac1^ neurons.

To assess the sufficiency of the Sp5C^Tac1^‐PBN pathway in orofacial pain modulation, we injected AAV2/9‐DIO‐ChR2‐EYFP virus into the right Sp5C of *Tac1*‐*Cre* mice and implanted optical fibers into the right PBN for pathway‐specific activation, with AAV2/9‐DIO‐EYFP virus as a control (Figure 4S; Figure S7A). We found that optogenetic stimulation significantly increased wiping events in ChR2‐expressing mice during the light‐on period vs. light‐off periods, and vs. the control group (Figure 4T,U). Consistently, ChR2‐expressing mice exhibited significantly shorter wiping latencies to laser‐on compared to EYFP controls (Figure S7B). Moreover, mechanical withdrawal thresholds observed in the ChR2 group were significantly reduced during stimulation vs. non‐stimulation intervals, and vs. EYFP controls (Figure 4V). To evaluate whether pain‐induced aversion could be induced by activating the Sp5C^Tac1^‐PBN pathway, we performed the RTPA test (Figure S7C). Our data revealed that ChR2‐expressing mice spent significantly less time in the optogenetic stimulation‐paired chamber than EYFP controls (Figure S7D,E). Collectively, the Sp5C^Tac1^‐PBN pathway manipulation demonstrates that pathway activation is sufficient to drive TN‐related nociceptive behaviors and aversion.

### 2.5 *Tac1* Gene Expressed in PBN‐Projecting Sp5C Neurons Modulate TN Development

**FIGURE 5:**
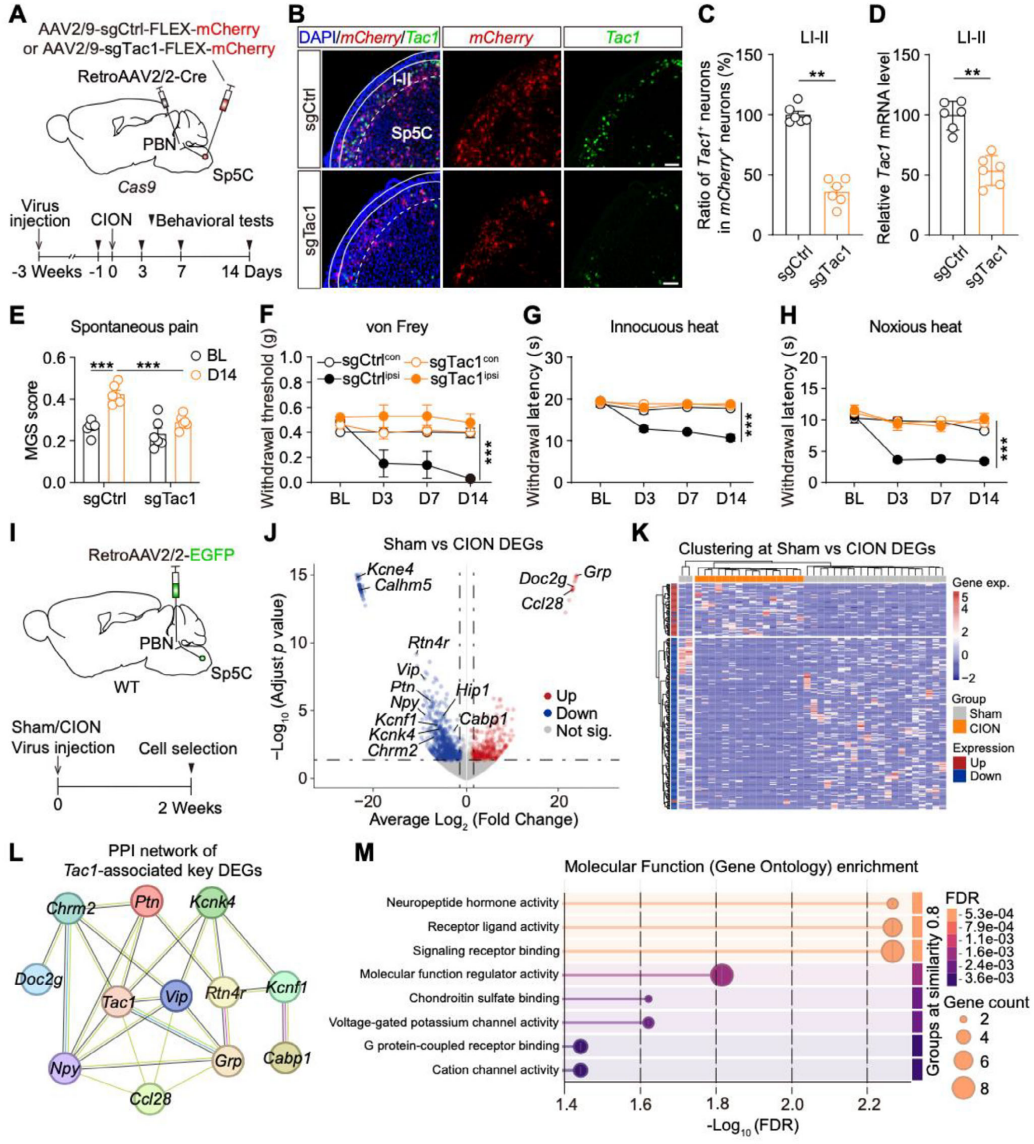
*Tac1* gene expressed in Sp5C neurons crucially modulates TN. (A), A schematic and timeline for the experiment. (B), Example images showing *mCherry* (red) and *Tac1* (green) gene expression patterns in the right Sp5C from mice in both groups on D14 after CION. Blue: DAPI. Scale bar: 100 µm. (C), Percentages of *Tac1*‐positive neurons in *mCherry*‐positive neurons in the right Sp5C from mice in both groups. ^**^ *p* < 0.01, by Mann Whitney test. (D), Relative *Tac1* gene expression in *Tac1*‐positive Sp5C neurons from mice in both groups. ^**^ *p* < 0.01, by Mann Whitney test. (E–H), MGS score (E), withdrawal threshold (F), and withdrawal latency to innocuous heat (G) and noxious heat (H) measured in mice from both groups. ^***^ *p* < 0.001, by two‐way ANOVA (n = 6 mice for each group). (I), A schematic and timeline for the experiment. (J), A volcano plot showing DEGs detected in *Tac1*‐positive PBN‐projecting Sp5C neurons from mice in the sham and CION groups. Log_2_ (Fold Change) > 1 or Log_2_ (Fold Change) < ‐1, and adjusted *p* < 0.05 (n = 39 neurons from 6 mice). (K), A heatmap of DEGs detected in *Tac1*‐positive PBN‐projecting Sp5C neurons from mice in both groups. (L), PPI network of key DEGs with a minimum required interaction score set to a low confidence level of 0.15. PPI enrichment p value of the network was < 8.6e–12. (M), Molecular Function (Gene Ontology) enrichment analysis for key DEGs between both groups.

The *Tac1* gene encodes substance P (SP), a neuropeptide implicated in corneal pain modulation [42]. We therefore investigated whether the *Tac1* gene expressed in PBN‐projecting Sp5C neurons is involved in TN modulation. To address this, we genetically knocked‐down *Tac1* gene in the PBN‐projecting Sp5C neurons by injecting RetroAAV2/2‐Cre virus into the right PBN and AAV2/9‐sgTac1‐FLEX‐mCherry virus into the right Sp5C of *Cas9* mice (which express Cas9 protein in a Cre‐dependent manner), with AAV2/9‐sgCtrl‐FLEX‐mCherry virus as a control (Figure 5A). The *Tac1* gene knockdown efficiency was validated by quantifying the *Tac1* expression via RNAscope ISH in virally infected neurons of the Sp5C. We found that compared with controls, ratio of *Tac1‐*positive neurons was significantly lower in the sgTac1‐infected neurons (Figure 5B,C). Moreover, the relative *Tac1* mRNA level in *Tac1*‐positive neurons was markedly lower in the sgTac1 group vs. control group (Figure 5B,D). These data confirmed efficient Tac1 knockdown in PBN‐projecting Sp5C neurons. Three weeks after virus expressions, we did baseline behavioral tests and revealed no significant difference in orofacial spontaneous responses and mechanical/thermal pain threshold between the two groups (Figure 5E–H). These data indicate *Tac1* expression in PBN‐projecting Sp5C neurons does not modulate acute orofacial nociception. Subsequently, we performed the CION surgery, and assessed the pain‐related behaviors on distinct days. Results revealed that Tac1 knockdown in PBN‐projecting Sp5C neurons significantly alleviated the TN‐induced pain hypersensitivity, indicated by a decrease in MGS score (Figure 5E), and an increase in mechanical withdrawal threshold (Figure 5F) and thermal withdrawal latency (Figure 5G,H). These data suggest *Tac1* gene expressed in PBN‐projecting Sp5C neurons are essential for TN development.

Beyond its role in orofacial nociception, Sp5C has been implicated in grooming behavior modulation [5]. To determine whether *Tac1* expression in PBN‐projecting Sp5C neurons regulates grooming behaviors, we quantified oil‐induced grooming behaviors for mice in both groups. No significant differences were observed in both the grooming‐like wiping events under the head‐fixed state (Figure S7F–H) and grooming duration under the free‐moving state (Figure S7I–K). These data demonstrate that the *Tac1* expression in PBN‐projecting Sp5C neurons is dispensable for grooming behaviors.

Given that *Tac1*‐positive neurons constitute the predominant neuronal population in PBN‐projecting Sp5C neurons, we investigated whether TN induces transcriptional alterations in *Tac1*‐positive PBN‐projecting Sp5C neurons. After retrograde labeling PBN‐projecting Sp5C neurons with EGFP (Figure 5I), we manually picked EGFP‐positive cells from mice in the sham and CION groups, constructed Smart‐seq3 libraries, and performed RNA sequencing using the previously established protocol (Figure 3B). We identified *Tac1*‐positive neurons using the same criteria (Log_2_ (TPM+1) > 6) as in Figure 3D, and did all subsequent transcriptome analyses on this defined population. Differentially expressed genes (DEGs) with a fold change greater than 2 and a *p* value less than 0.05 were selected based on the sequencing results (Figure 5J; Figure S8A). We identified 63 up‐regulated and 212 down‐regulated DEGs in *Tac1*‐positive neurons between the sham and CION groups. Subsequent gene function analysis indicate 14 genes may associated with pain, neuronal excitability, and synaptic transmission, including 3 up‐regulated genes *gastrin‐releasing peptide* (*Grp*), *double C2 protein* (*Doc2g)* [43], *CC chemokine 28* (*Ccl28*) [44], and 11 down‐regulated genes *vasoactive intestinal polypeptide* (*Vip*), *neuropeptide Y* (*Npy*) [45], *potassium voltage‐gated channel, subfamily F member 1* (*Kcnf1*) [46], *potassium channel, subfamily K, member 4* (*Kcnk4*) [47], *potassium voltage‐gated channel, Isk‐related subfamily, gene 4* (*Kcne4*) [48], *calcium homeostasis modulator family member 5* (*Calhm5*) [49], *calcium binding protein 1* (*Cabp1*) [50], *reticulon 4 receptor* (*Rtn4r*) [51], *pleiotrophin* (*Ptn*) [52], *huntingtin interacting protein 1* (*Hip1*) [53], and *M2 muscarinic cholinergic receptors* (*Chrm2*) [54]. Collectively, these transcriptional alterations revealed CION‐induced rewiring of pain‐signal transmission and synaptic functions (Figure 5J,K). We further constructed the protein‐protein interaction (PPI) network of Tac1‐associated key DEGs, and mapped both functional and physical protein associations (Figure 5L). Overall, DEGs we mentioned above were involved in pathways related to neuropeptide hormone activity, voltage‐gated potassium channel activity, and neuroactive ligand receptor interaction (Figure 5M; Figure S8B–D). Taken together, these data demonstrate transcriptional reprogramming induced by CION in PBN‐projecting Sp5C^Tac1^ neurons, indicating underlying molecular mechanisms of TN pathology.

### 2.6 Sp5C^Tac1^ Neurons Directly Relay TG Signals to PBN

**FIGURE 6:**
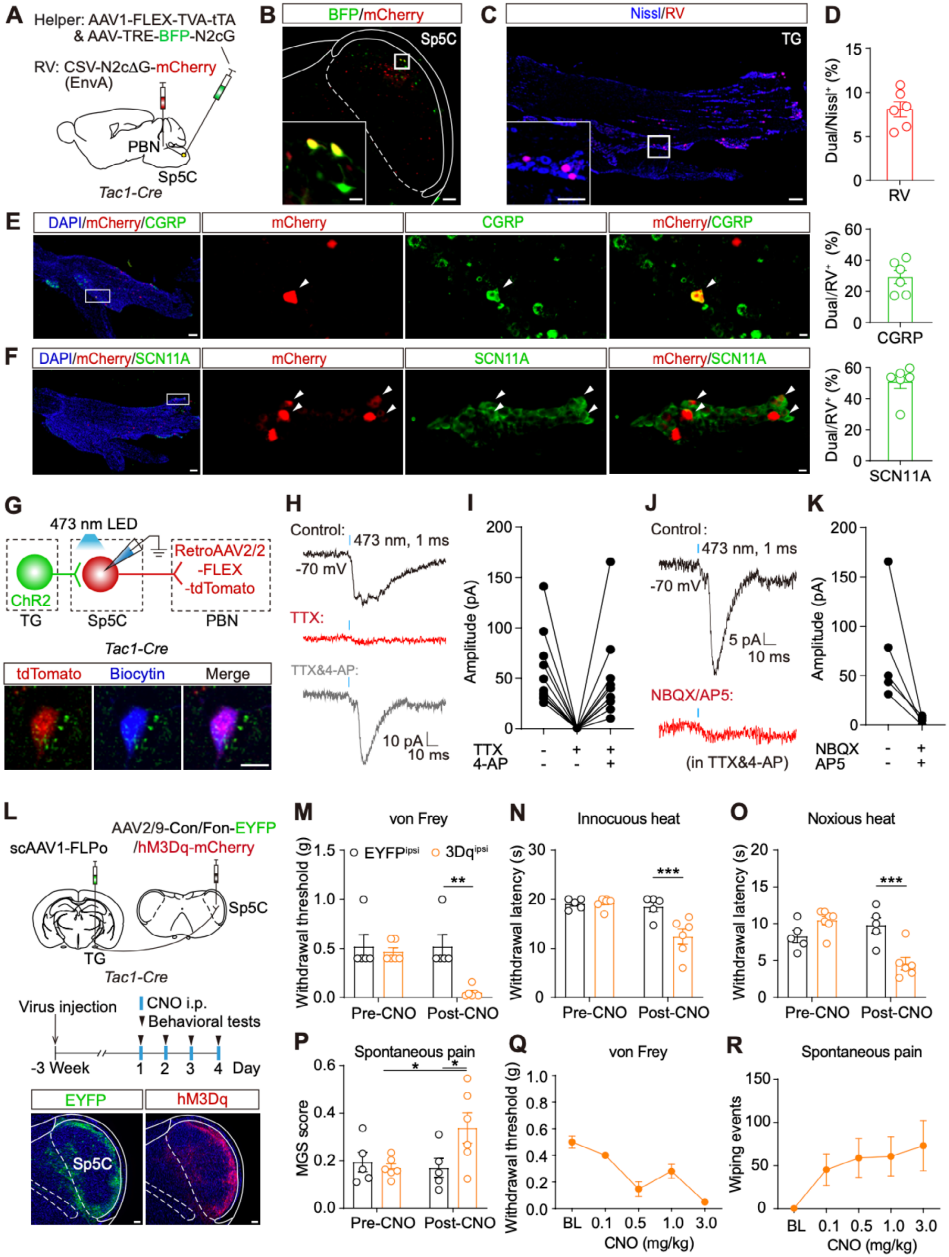
Sp5C^Tac1^ neurons receive direct inputs from TG neurons. (A), A schematic for the rabies virus‐based retrograde tracing. (B), An example of a virus expression pattern in the right Sp5C. Green: BFP, red: mCherry. Scale bar: 100 µm. Inset scale bar: 20 µm. (C), Distribution pattern of mCherry‐positive neurons in the right TG. Red: mCherry, blue: Nissl. Scale bar: 200 µm. Inset scale bar: 20 µm. (D), Ratio of mCherry‐positive neurons in Nissl positive neurons measured in the right TG. Dual: dual‐labeled. (E‐F), Distribution pattern of mCherry and CGRP‐ (E)/SCN11‐ (F) positive neurons in right TG (left). Ratio of CGPR‐/SCN11A‐positive neurons in RV‐expressed neurons (right). Red: mCherry, green: CGRP/SCN11A, blue: DAPI. Scale bar: 200 µm. Inset scale bar: 20 µm. (G), A schematic for electrophysiological recording. Images showing an example of a recorded tdTomato‐labeled PBN‐projecting Sp5C^Tac1^ neuron. Red: PBN‐projecting Sp5C^Tac1^ neurons; Blue: biocytin. Scale bar: 10 µm. (H), Examples of light‐evoked EPSCs recorded from PBN‐projecting Sp5C^Tac1^ neurons at baseline (black), after application of TTX (red), and further addition of 4‐AP (gray). (I), Amplitudes of light‐evoked EPSCs recorded under distinct conditions (n = 10 neurons from 6 mice). (J), Examples of light‐evoked EPSCs recorded from PBN‐projecting Sp5C^Tac1^ neurons at baseline (black) and after application of NBQX/AP5 (red) in the presence of TTX/4‐AP. (K), Amplitudes of light‐evoked EPSCs recorded without and with NBQX/AP5 (n = 5 neurons from 3 mice). (L), Schematic and timeline for chemogenetic activation of Sp5C^Tac1^ neurons, which receive TG inputs. Images showing examples of EYFP and hM3Dq virus expression pattern in Sp5C. Scale bar: 100 µm. (M–P), Mechanical withdrawal responses to von Frey filaments (M), withdrawal latency to innocuous heat (N) and noxious heat (O), and spontaneous pain (P) were measured before and after CNO application in both groups of mice. ^*^ *p* < 0.05, ^****^ *p* < 0.0001, by two‐way ANOVA (EGFP, n = 5; hM3Dq, n = 6). (Q,R), Mechanical threshold (Q) and wiping events (R) on the ipsilateral side were measured following the injection of different CNO concentrations in hM3Dq‐injected mice (n = 6).

To determine whether PBN‐projecting Sp5C^Tac1^ neurons receive direct TG inputs, we employed RV‐based trans‐synaptic tracing. *Tac1*‐*Cre* mice received the injection of helper viruses, AAV1‐FLEX‐split TVA‐P2A‐EGFP‐P2A‐tTA and AAV‐TREtight‐mTagBFP2‐ N2cG, into the right Sp5C. Seven days post helper virus injection, we injected CSV‐N2ΔG‐mCherry (EnvA) virus into the right PBN to retrogradely label PBN‐projecting Sp5C^Tac1^ neurons and their presynaptic inputs (Figure 6A,B). Another seven days after the RV injection, we observed mCherry‐positive neurons in the right TG (Figure 6C,D), indicating PBN‐projecting Sp5C^Tac1^ neurons receive direct synaptic inputs from TG. To characterize these RV‐labeled TG neurons, we performed immunochemistry staining to examine the colocalization of RV with markers of noxious (CGRP, SCN11A, IB4) and innocuous (NF200) nociceptors (Figure 6E,F; Figure S9A,B). Quantification revealed that among all RV‐positive TG neurons, 29.2% were CGRP‐positive (Figure 6E), 50.8% were SCN11A‐positive (Figure 6F), 5.6% were IB4‐positive (Figure S9A), and 40.4% were NF200‐positive (Figure S9B). These data demonstrated that TG transmits both noxious and innocuous sensory signals to PBN‐projecting Sp5C^Tac1^ neurons. Additionally, RV‐labeled neurons were detected in descending modulatory regions, including the motor cortex, the somatosensory cortex, the red nucleus (magnocellular part), and the raphe magnus nucleus (Figure S9C).

We further verified the functional monosynaptic connections between TG and PBN‐projecting Sp5C^Tac1^ neurons by utilizing an optogenetic‐assisted electrophysiology approach. *Tac1‐Cre* mice received AAV2/9‐ChR2‐EGFP virus injection into the right TG to express ChR2 in TG neurons, and RetroAAV2/2‐FLEX‐tdTomato virus injection into the right PBN to express tdTomato in PBN‐projecting Sp5C^Tac1^ neurons (Figure 6G). Three weeks after virus expression, we did whole‐cell recordings from tdTomato‐positive PBN‐projecting Sp5C^Tac1^ neurons and recorded light‐evoked inward currents (Figure 6G). We found that, in a subset of neurons, these currents were blocked by TTX and restored by further application of 4‐AP, suggesting monosynaptic connections (Figure 6H,I). Quantification across all responsive PBN‐projecting Sp5C neurons revealed that 37% of neurons received monosynaptic inputs from TG, while 63% received polysynaptic inputs (Figure S9D). Moreover, these monosynaptic currents were blocked by NBQX/AP5, suggesting excitatory connections (Figure 6J,K). Taken together, these data suggest a subset of Sp5C^Tac1^ neurons directly relay excitatory TG signals to the PBN.

We then tested whether TG‐targeting Sp5C^Tac1^ neurons are sufficient to trigger orofacial pain behaviors. To selectively activate this population, we injected AAV1‐FLPo into the TG (marked by CTB‐488, Figure S9E) and AAV2/9‐Con/Fon‐hM3Dq‐mCherry into the Sp5C of the *Tac1‐Cre* mice. The AAV2/9‐Con/Fon‐EYFP was injected as a control. After the virus expression for 3 weeks, we administrated CNO and assessed behaviors (Figure 6L; Figure S9F). We found that in contrast to the stable thresholds in the EYFP‐injected control group, activation of TG‐projecting Sp5C^Tac1^ neurons significantly induced hyperalgesia. This was evidenced by a decreased mechanical threshold (Figure 6M), shortened thermal withdraw latency (Figure 6N,O), and an increased MGS score (Figure 6P). Furthermore, a dose‐dependent relationship was observed: higher CNO concentration led to a greater decrease in the mechanical threshold (Figure 6Q) and concomitant increase in spontaneous wiping events (Figure 6R). This data suggests TG targeting Sp5C^Tac1^ neurons are sufficient to drive nociceptive behaviors.

Together, our data demonstrate that PBN‐projecting Sp5C^Tac1^ neurons receive direct monosynaptic inputs from TG, and the TG‐Sp5C^Tac1^ pathway play sufficient role in orofacial pain processing.

## 3 Discussion

Our study identifies a cell‐type‐specific Sp5C^Tac1^‐PBN^Tac1^ pathway that critically modulates TN‐induced orofacial pain hypersensitivity. Although previous studies revealed the crucial role of Tac1 positive spinal and PBN neurons in pain and affective processing [13, 28, 39], our work extends the function of PBN^Tac1^ neurons within the trigeminal pain system.

### 3.1 The Contribution of PBN‐Projecting Sp5C^Tac1^ Neurons to TN Pathogenesis

The Sp5C has long been recognized as a key hub for orofacial pain processing, relaying TG signals to multiple downstream brain areas [2] and exhibiting increased neuronal activity during orofacial pain states [55]. However, the molecular markers for these projection neurons remain largely unidentified. Previous studies have demonstrated that PBN‐projecting Sp5C neurons co‐express *Tacr1* and *Oprm1* [16, 56]. We performed single‐neuron sequencing on PBN‐projecting Sp5C neurons, and detected significantly higher expression level of *Tac1*, compared to *Oprm1* and other identified marker genes for spinal projection neurons (Figure 3C). Additionally, our neural tracing results demonstrated that Sp5C^Tac1^ neurons sent dense projections to PBN. Collectively, these data proved that *Tac1* is the molecular marker for PBN‐projecting Sp5C neurons. At the behavioral level, we observed that activation of Sp5C^Tac1^‐PBN pathway induced more wiping behaviors with shorter latency, and higher aversive responses compared with activation of the non‐cell‐type‐specific Sp5C‐PBN pathway. Consistently, both excitatory and inhibitory projections were detected from Sp5C to PBN, while only excitatory connections were detected in the Sp5C^Tac1^‐PBN pathway. These findings suggest distinct functional roles of *Tac1‐*negative and *Tac1*‐positive PBN‐projecting Sp5C neurons in orofacial pain modulation. Notably, except for *Tac1* gene, our results demonstrated that *Nmur2* and *Lypd1* genes also expressed in PBN‐projecting Sp5C neurons. Their functional roles in TN modulation warrant further investigation. Beyond projection neurons, local inhibitory and excitatory neurons can also contribute to TN modulation. Previous studies have revealed the down‐regulation of CGRP in Sp5C attenuates migraine [57] and the neuronal activity of GABA_B_ neurons is elevated in TN models [58]. Our viral tracing data illustrated that *Pdyn*‐positive Sp5C neurons do not project to the PBN, suggest these neurons are local neurons. Previous study reported that inhibition or activation of these *Pdyn*‐positive local neurons bidirectionally modulates orofacial pain processing [59]. A critical future direction is to elucidate how these interneurons interact with PBN‐projecting Sp5C^Tac1^ neurons to fully unravel the circuit mechanisms underlying TN modulation.

The *Tac1* gene encodes SP, a neuropeptide well‐documented for its role in modulating somatic nociceptive transmission and the development of neuropathic pain [60, 61, 62]. In the context of orofacial pain, single‐cell RNA sequencing of TG has confirmed *Tac1* gene expression in both mice and humans [63]. Our study further demonstrates that *Tac1* gene is the major marker gene for PBN‐projecting Sp5C neurons. Specifically, knockdown *Tac1* gene in PBN‐projecting Sp5C neurons significantly attenuates TN‐induced pain hypersensitivity, establishing the critical role of SP in orofacial nociception. Although the expression of *Tacr1* (encodes a typical receptor for SP, NK1R) is low in the PBel ‐across neurons, microglia [64] and astrocytes [65] ‐ SP released by Sp5C^Tac1^ neurons may influence PBN^Tac1^ neurons through these local PBN^Tacr1^ cells. Additionally, NK1R‐positive axons have been defined in the PBel. Therefore, SP released from Sp5C^Tac1^ neurons may also influence PBN^Tac1^ neurons by acting on these presynaptic NK1R positive axons. Moreover, Allen Mouse Brain Atlas displayed that PBN neurons express Cav1.2 and HCN1, which could be activated through association with SP based on the IntAct Molecular Interaction Database. However, the mechanism by which SP released from Sp5C^Tac1^ neurons modulates PBN^Tac1^ neurons requires further investigation.

Current first‐line pharmacotherapy for TN primarily acts through non‐specific mechanisms such as voltage‐gated sodium channel inhibitors (e.g., carbamazepine or oxcarbazepine) and GABA agonists (e.g., baclofen), often causing adverse effects including dizziness, ataxia, and liver dysfunction. Through transcriptomic profiling of PBN‐projecting Sp5C^Tac1^ neurons from both sham and CION mice, we identified significant dysregulation of multiple genes (63 up‐regulated and 212 down‐regulated) related to pain and synaptic function in CION models. Specifically, we identified the CION‐induced dysregulation of genes encoding sex hormone receptors, which may underlie the sexual difference for TN development. Although the functional implications for these changes require further investigation, the promising candidate pathways revealed by our sequencing data open new avenues for the development of target‐specific therapies.

### 3.2 Critical Roles of PBN^Tac1^ Neurons in TN Pathogenesis

The PBN serves as a critical hub for processing nociceptive signals from multiple origins, including face, body, and visceral organs [24]. Although the molecular and circuit mechanisms for the involvement of PBN in somatic pain processing, both acute and chronic, have been extensively characterized [24, 66, 67], the neural mechanisms mediating facial pain remain largely unknown. Our study identified that PBN^Tac1^ neurons receive projections from Sp5C and respond to noxious mechanical and thermal stimuli. In the CION model, the noxious stimuli‐evoked responses in these neurons increased, but this increase did not reach statistical significance. This is likely attributable to a near‐ceiling level of baseline activity. Using a chemogenetic approach, we further revealed the crucial role of these neurons in TN modulation. This finding aligns with previous studies demonstrating that facial noxious stimuli activate PBN neurons [21, 22], and activation of PBN‐VTA pathway reverses TN‐induced depression [23]. Except for PBN, Sp5C neurons sent projections to other brain areas, including thalamus, periaqueductal gray (PAG), and hypothalamus. Although we found that inhibition of PBN^Tac1^ neurons attenuated the TN‐induced pain hypersensitivity, the onset time for pain relief in PBN^Tac1^ neurons inhibition experiment (7 days) is later than PBN‐projecting Sp5C neurons inhibition experiment (3 days). These data suggest the potential involvement of additional Sp5C ascending pathways in the modulation of TN onset, warranting further investigation.

Anatomically, PBN^Tac1^ neurons are predominantly located in the PBel [38]. Although PBel specifically receives orofacial pain signals, it comprises diverse cell types, including those expressing *Calca*, *Chat*, *Oprm1*, *Crhr1* and *Tac1* [38]. In the present study, we investigated the roles of *Tac1‐* and *Oprm1‐*expression neurons in TN modulation and found *Tac1‐*positive neurons, rather than *Oprm1‐*positive neurons, play crucial roles in TN modulation. Oprm1 represents a relatively abundant cell type in the PBel, encompassing two functionally distinct neuronal populations—33% are PBN^Tac1^ neurons and 19% are PBN^CGRP^ neurons [39]—which exhibit opposing effects. Although *Calca*‐positive PBN (PBN^Calca^) neurons exhibit overlapping expression with *Tac1* [38, 68], literature found that activation of PBN^Tac1^ neurons and PBN^CGRP^ neurons in the PBel leads to opposite defensive behavioral responses, including locomotion and freezing, due to the distinct axonal terminal distribution regions in their downstream brain areas [68]. This heterogeneity explains why chemogenetic inhibition of PBN^Oprm1^ did not yield the same pain‐related behavioral phenotypes as observed with PBN^Tac1^ inhibition. Except for the role in pain processing, previous studies reported that the activation of PBN^Tac1^ neurons increases [39] and the inhibition of PBN^Oprm1^ neurons decreases the breath rate [69]. In this study, we found that the inhibition of PBN^Tac1^ neurons has no effect on breathing, suggest the unnecessary role of PBN^Tac1^ neurons in breath control. The suppression of breathing following PBN^Oprm1^ neuron inhibition may be mediated by Oprm1^+^/Tac1^−^ neurons. Additionally, whether other types of PBel‐located neurons are involved in orofacial pain processing remains to be explored.

PBN^Tac1^ neurons have been reported to modulate somatic pain‐induced escape behaviors via a descending projection to the medullary reticular nucleus (MdD) [30]. Additionally, activation of PBN^Tac1^ neurons elicits both coping behaviors and affective aversion [68]. Moreover, the application of SP in PBN has been shown to shape the morphology of vocalization [70]. These studies highlight the crucial role of PBN^Tac1^ neurons in both sensory and affective pain processing. Consistently, we found that activation of the Sp5C^Tac1^‐PBN pathway induced aversion. While for PBN^Tac1^ neurons, we only tested the role of these neurons in modulating TN‐induced pain hypersensitivity, whether they also get involved in TN‐induced anxiety‐ or depression‐like behaviors needs further investigation.

### 3.3 The Direct TG‐PBN Pathway and the Indirect TG‐Sp5C‐PBN Pathway

The PBN receives TG inputs through both the direct TG‐PBN pathway [6], and indirect TG‐Sp5C‐PBN pathway [2]. We focused on PBN^Tac1^ neurons and found that these neurons mainly receive indirect inputs from the Sp5C rather than direct input from the TG. Thus, we conclude that PBN^Tac1^ neurons predominantly receive indirect TG inputs via the Sp5C. This indirect pathway was further confirmed through retrograde tracing of PBN‐projecting Sp5C^Tac1^ neurons and recording of monosynaptic connections between TG and PBN‐projecting Sp5C^Tac1^ neurons. Moreover, in the TN model, the excitatory inputs to PBN‐projecting Sp5C^Tac1^ neurons and the connectivity between Sp5C^Tac1^ and PBN^Tac1^ neurons were significantly increased compared with the control group. These results suggest TN gives rise to plasticity of this indirect pathway. Consistent with these findings, our behavioral results illustrated that the Sp5C‐PBN pathway was critically involved in TN modulation.

In addition to the indirect pathway, previous studies have reported the involvement of the direct TG‐PBN pathway in orofacial pain‐related emotion processing [6]. Although the direct TG‐PBN^Tac1^ pathway is rare, as we shown in this study, it may also play roles in TN modulation, warranting investigation in the future. Moreover, except for PBN^Tac1^ neurons, the PBN contains other types of neurons. Further studies are required to define which specific type of PBN neurons receive direct TG inputs, and to elucidate their roles in orofacial pain processing. Additionally, it would be valuable to investigate whether the direct and indirect pathways interact with each other or whether these two pathways play distinct roles in orofacial pain processing in the future.

### 3.4 Distinct Mechanisms Underlie the Processing of Orofacial and Somatic Pain

Distinct from somatic pain processing, the circuit mechanisms underlying orofacial pain processing remain largely unknown. In this study, we systematically delineated the cellular and circuitry mechanisms involved in orofacial pain processing and discovered fundamentally different mechanisms underlying somatic and orofacial pain processing. At the Sp5C level, we found that the *Tac1* gene, rather than the *Tacr1* gene, serves as the major molecular marker for PBN‐projecting neurons. However, in the spinal cord, both *Tac1‐* and *Tacr1‐*positive neurons are subtypes of spinal projection neurons [11, 12, 13, 71]. The differential properties of *Tacr1‐*positive neurons in the spinal cord and the Sp5C suggest fundamentally distinct mechanisms governing somatic vs. orofacial pain processing.

At the PBN level, we discovered that *Tac1‐*positive PBel neurons are involved in orofacial pain processing. In contrast, *Tacr1* neurons localized in the dorsolateral PBN (PBdl) have been reported to play crucial roles in somatic acute and chronic pain processing [38, 72, 73]. Moreover, previous study reported that inhibition of PBel‐located PBN^Tac1^ neurons has no effect on somatic neuropathic pain‐induced hypersensitivity [73]. These findings indicate that somatic and orofacial pain processing involves distinct PBN cell types. Except for distinct cell types, tracing results show that the PBel and PBdl subareas exhibit markedly different whole‐brain projection patterns [38, 67], suggesting potential divergent circuit mechanisms underlying somatic vs. facial pain processing. Given that orofacial pain typically induces a stronger emotional response compared to somatic pain, elucidating the circuit mechanism underlying this phenomenon could be of significant importance in future research. However, the existence of connections between the PBdl‐PBel may enable shared circuit mechanisms for both somatic and facial pain processing [67]. Supporting this hypothesis, higher‐order brain regions, including the anterior cingulate cortex (ACC), the prefrontal cortex (PFC); and a critical brain area in the descending pathway, the PAG, have been implicated involving in both somatic and facial pain processing [2].

## 4 Materials and Methods

### 4.1 Animals

Male and female mice (8–10 weeks old) were used in this study. C57BL/6J mice and *Cas9* mice were purchased from Gempharmatech Co., Ltd. (Jiangsu, China). The *Tac1‐Cre*, *Pdyn‐Cre* and *Oprm1‐CreER* mouse lines were kindly provided by Dr. Rong‐Feng Hu's and Dr. Shu‐Min Duan's laboratory, respectively, at the Institute of Translational Brain Research, Fudan University. The *Tacr1‐FLP*o [72] mouse line and *Nmur2‐Cre* [74] mouse line were gifts from Dr. Yan‐Gang Sun's laboratory at the Chinese Academy of Sciences (Shanghai, China). Mice were housed under standard conditions with a 12‐h light ‐ dark cycle and ad libitum access to water and food. All experiments were approved by the Fudan University Animal Care and Use Committee (Approval No. 202105008S) and conducted in accordance with the ethical guidelines of the National Institutes of Health and the International Association for the Study of Pain. Every effort was made to minimize animal suffering and reduce the number of animals used.

### 4.2 Trigeminal Neuropathic Pain Model

Chronic constriction injury of the infraorbital nerve (CION) was performed to induce trigeminal neuropathic pain in mice, as previously described [32, 33]. Briefly, mice were anesthetized via i.p. administration of a cocktail of Zoletil (Tiletamine hydrochloride: 12.5 mg/kg) and Rompun (Xylazine hydrochloride: 0.08 mg/kg), and reflex absence was confirmed prior to surgery. Using sterile technique, a 2‐mm incision was made in the palatal buccal mucosa. The right infraorbital nerve was then exposed via a blunt dissection and loosely ligated with a 5‐0 chromic gut suture (Shandong Haidike Medical Products). Sham control mice underwent identical surgical exposure of the nerve without ligation. Postoperatively, mice recovered in a clean box on a heating pad. To manage pain and inflammation, tolfenamic acid (0.04 mL/kg, i.p.) and benzylpenicillin sodium (477.6 mg/kg, i.p.) were administered daily for three consecutive days, beginning immediately after surgery. Given that CION–induced neuropathic pain persists at least 28 days and reaches a stable plateau within 14 days post‐surgery, all subsequent pain‐related behavioral tests were conducted during this initial 14‐day period. Animals were allocated in sham and CION groups randomly. Animal age and gender were included as blocking variables in the study designs prior to experimental assessment.

### 4.3 Stereotaxic Injection

Mice were anesthetized via i.p. administration of Zoletil (Tiletamine hydrochloride: 12.5 mg/kg) and Rompun (Xylazine hydrochloride: 0.08 mg/kg) cocktail. Reflex absence was confirmed prior to surgery. Ocular protection was ensured by bilateral application of lubricating ophthalmic ointment. A midline scalp incision exposed the skull, and a small craniotomy was created over the target brain region using a hand‐held electric drill.

Virus injections were performed using a glass pipette attached to a Nanoliter Microinjection Pump (R‐480, RWD Life Science) at a flow rate of 1 nL/s. Following injection, the pipette remained in situ for 5 min before slow withdrawal.

Stereotaxic coordinates relative to bregma were as follows:

PBN: Anteroposterior (AP): ‐5.35 mm, Mediolateral (ML): ‐1.68 mm, Dorsoventral (DV): ‐3.68 mm.

TG: AP: ‐1.80 mm, ML: ‐2.00 mm, DV: ‐6.40 mm.

For virus injection in the Sp5C:

The neck was immobilized with ear rods, and the foramen magnum was exposed using muscle retractors. After a dural incision, a glass pipette was angled 30° rightward and inserted at the coordinates: ML: ‐1.05 mm, DV: ‐0.30 mm, with the rostrocaudal level aligned to the transverse midline of the exposed area.

Postoperatively, mice recovered on a heating pad before returning to home cages. A minimum 3‐week recovery was allowed before experimentation. Viral titers listed below are expressed as the number of viral genome copies per milliliter (v.g./mL).

To examine the activity of PBN^Tac1^ neurons with fiber photometry, AAV2/9‐hEF1a‐DIO‐GCaMP6s‐WPRE‐pA virus (3.67 × 10^12^) was injected into the right PBN of *Tac1‐Cre* mice. Optical fibers were implanted above the injection sites (coordinate: AP: ‐1.68 mm, ML: ‐5.35 mm, DV: ‐3.5 mm).

To chronically inhibit the activity of PBN^Tac1^ neurons for behavioral tests, AAV2/8‐hSyn‐DIO‐hM4D(Gi)‐EGFP‐WPRE‐pA virus (8.30 × 10^12^) was injected into the right PBN of *Tac1‐Cre* mice. AAV2/8‐hSyn‐DIO‐EGFP‐WPRE‐pA virus (4.00 × 10^12^) was injected as the control.

To chronically inhibit the activity of Sp5C ^Tac1^ neurons for behavioral tests, AAV2/9‐hEF1a‐DIO‐hM4D(Gi)‐mCherry‐WPRE‐pA virus (8.30 × 10^12^) was injected into the right Sp5C of *Tac1‐Cre* mice. AAV2/9‐hEF1a‐DIO‐EYFP (3.95 × 10^12^) was injected as the control.

To chronically inhibit the activity of PBN^Oprm1^ neurons for behavioral tests, AAV2/9‐ hEF1a‐DIO‐hM4D(Gi)‐mCherry‐WPRE‐pA virus (8.30 × 10^12^) was injected into the right PBN of *Oprm1‐CreER* mice. AAV2/9‐hEF1a‐DIO‐EYFP (3.95 × 10^12^) was injected as the control. 4‐OH tamoxifen (100 mg/kg in corn oil) was i.p. injected 1 week after viral injection every other day for 3 times.

To label the upstream brain areas of PBN^Tac1^ neurons, AAV1‐FLEX‐split TVA‐P2A‐EGFP‐P2A‐tTA virus [75] (4.19 × 10^12^) mixed with AAV‐TREtight‐mTagBFP2‐N2cG virus [76] (1.3 × 10^13^) were injected into the right PBN of *Tac1‐Cre* mice. One week later, CSV‐N2ΔG‐4mCherry (EnvA) virus [77] (1.0 × 10^9^ IU/mL) was injected into the same site.

To label the Sp5C neurons and PBN^Tac1^ neurons for ex vivo electrophysiological recordings, AAV2/9‐hSyn‐hChR2(H134R)‐mCherry‐WPRE‐pA virus (6.37 × 10^12^) was injected into the rSp5C and AAV2/9‐hEF1a‐DIO‐EYFP‐WPRE‐pA virus (3.95 × 10^12^) was injected in to the right PBN of *Tac1‐Cre* mice.

To chronically inhibit the PBN‐projecting Sp5C neurons, scAAV2/2Retro‐hSyn‐Cre‐pA virus (1.91 × 10^13^) was injected into the right PBN, and AAV2/9‐hEF1a‐DIO‐hM4D(Gi)‐mCherry‐ER2‐WPRE‐pA virus (4.00 × 10^12^) was injected into the right Sp5C of wild type mice. AAV2/9‐hEF1a‐DIO‐EYFP‐WPRE‐pA virus (3.95 × 10^12^) was injected into the right Sp5C as the control. To trace the outputs of Sp5C^Tac1/Pdyn/Nmur2^ neurons, AAV2/9‐hEF1a‐DIO‐EYFP‐WPRE‐pA virus (3.95 × 10^12^) was injected into the right Sp5C of *Tac1‐Cre* mice or *Pdyn‐Cre* mice or *Nmur2‐Cre* mice.

To trace the outputs of Sp5C^Tacr1^ neurons, AAV2/9‐hEF1a‐fDIO‐hM4D(Gi)‐mCherry‐WPRE‐pA virus (4.23 × 10^12^) was injected into the right Sp5C of *Tacr1‐FLPo* mice.

To trace the *Tac1*‐positive upstream brain areas of PBN neurons, AAV2/2 Retro plus‐hSyn‐FLEX‐tdTomato‐WPRE‐pA virus (1.57 × 10^13^) was injected into the right PBN of *Tac1‐Cre* mice.

To label the Sp5C^Tac1^ neurons and PBN^Tac1^ neurons for ex vivo electrophysiological recordings, AAV2/9‐hEF1a‐DIO‐hChR2(H134R)‐EYFP‐WPRE‐pA virus (3.34 × 10^12^) was injected into the right Sp5C, and AAV2/9‐hSyn‐DIO‐mCherry‐pA virus (3.28 × 10^12^) was injected into the right PBN of *Tac1‐Cre* mice.

To label the PBN‐projecting Sp5C^Tac1^ neurons for ex vivo electrophysiological recordings, AAV2/2Retro‐plus‐hSyn‐FLEX‐tdTomato‐WPRE‐pA virus (1.57 × 10^13^) was injected into the right PBN of *Tac1‐Cre* mice.

To examine the activity of Sp5C^Tac1^ neurons axon in PBN with fiber photometry, AAV2/9‐hEF1a‐DIO‐GCaMP6s‐WPRE‐pA virus (3.67 × 10^12^) were injected into the right Sp5C of *Tac1‐Cre* mice. Optical fibers were implanted in the right PBN (coordinate: AP: ‐1.68 mm, ML: ‐5.35 mm, DV: ‐3.55 mm).

To acutely activate the axons of Sp5C^Tac1^ neurons in PBN for behavioral tests, AAV2/9‐hEF1a‐DIO‐hChR2(H134R)‐EYFP‐WPRE‐pA virus (3.34 × 10^12^) was injected into the right Sp5C of *Tac1‐Cre* mice. AAV2/9‐hEF1a‐DIO‐EYFP‐WPRE‐pA virus (3.95 × 10^12^) was injected as the control. Optical fibers were implanted in the right PBN (coordinate: AP: ‐1.68 mm, ML: ‐5.35 mm, DV: ‐3.5 mm).

To knock down *Tac1* gene in PBN‐projecting Sp5C^Tac1^ neurons for behavioral tests, scAAV2/2Retro‐hSyn‐Cre‐pA virus (1.91 × 10^13^) was injected into the right PBN, and pAAV‐U6‐sgTac1‐3‐U6‐sgTac1‐4‐CAG‐FLEX‐mCherry virus (4.22 × 10^13^) was injected into the right Sp5C of *Cas9* mice. pAAV‐U6‐sgCtrl‐CAG‐FLEX‐mCherry‐WPRE virus (4.71 × 10^13^) was injected into the right Sp5C as the control.

To chronically activate the Sp5C^Tac1^ neurons which receive TG inputs, scAAV2/1‐hSyn‐FLPo‐pA (1.10 × 10^13^) mixed with CTB‐488 (0.01 mg/mL) was injected into the right TG, and AAV2/9‐hsyn‐Con/Fon‐hM3D(Gq)‐mCherry‐WPRE‐pA (2.19 × 10^13^) was injected into the right Sp5C of *Tac1‐Cre* mice. AAV2/9‐hSyn‐Con/Fon‐EYFP (5.00 × 10^12^) was injected into the right Sp5C as the control.

To label the upstream brain areas of PBN‐projecting Sp5C^Tac1^ neurons, AAV1‐FLEX‐split TVA‐P2A‐EGFP‐P2A‐tTA virus (1.4 × 10^13^) mixed with AAV‐TREtight‐mTagBFP2‐N2cG virus (1.3 × 10^13^) were injected into the right Sp5C of *Tac1‐Cre* mice. One week later, CSV‐N2ΔG‐4mCherry (EnvA) virus (1.0 × 10^9^) was injected into the right PBN.

To label the TG neurons and PBN‐projecting Sp5C ^Tac1^ neurons for ex vivo electrophysiological recordings, AAV2/9‐hSyn‐hChR2(H134R)‐EYFP‐WPRE‐pA virus (1.16 × 10^12^) was injected into the right TG and AAV2/2‐Retro‐plus‐hSyn‐FLEX‐tdTomato‐WPRE‐pA virus (1.57 × 10^13^) was injected into the right PBN of *Tac1‐Cre* mice.

To chronically inhibit the activity of PBN neurons or Sp5C neurons for behavioral tests, AAV2/8‐hSyn‐hM4D(Gi)‐mCherry‐WPRE‐pA virus (3.13 × 10^12^) was injected into the right PBN or Sp5C of wild type mice. AAV2/8‐hSyn‐EGFP‐WPRE‐pA virus (3.67 × 10^12^) was injected as the control.

To trace the upstream brain areas of PBN, CTB555 (1 mg/mL) was injected into the right PBN of wild type mice.

To trace the downstream brain areas of Sp5C, AAV2/9‐hSyn‐EGFP.PreSynapse‐WPRE‐pA virus (2.62 × 10^12^) was injected into the right Sp5C of wild type mice.

To examine the activity of PBN‐projecting Sp5C neurons axon in PBN with fiber photometry, AAV2/9‐hSyn‐GCaMP6s‐WPRE‐pA virus (3.84 × 10^12^) was injected into the right Sp5C of wild type mice. Optical fibers were implanted in the right PBN (coordinate: AP: ‐1.68 mm, ML: ‐5.35 mm, DV: ‐3.55 mm).

To acutely activate Sp5C neurons axons in PBN for behavioral tests, scAAV2/2Retro‐hSyn‐Cre‐pA virus (1.91 × 10^13^) was injected into the right PBN, and AAV2/9‐hEF1a‐DIO‐hChR2(H134R)‐EYFP‐WPRE‐pA virus (3.34 × 10^12^) was injected into the right Sp5C of wild type mice. AAV2/9‐hEF1a‐DIO‐EYFP‐WPRE‐pA virus (3.95 × 10^12^) was injected in the right Sp5C as the control. Optical fibers were implanted in the right PBN (coordinate: AP: ‐1.68 mm, ML: ‐5.35 mm, DV: ‐3.5 mm).

To label the PBN‐projecting Sp5C neurons for ex vivo electrophysiological recordings, AAV2/2Retro‐hSyn‐mCherry‐3Flag‐WPRE‐pA virus (2.26 × 10^12^) was injected into the right PBN of wild type mice.

To label the PBN‐projecting Sp5C neurons for single‐cell sequencing, AAV2/2Retro‐hSyn‐EGFP(‐3Flag)‐WPRE‐pA virus (1.12 × 10^13^) was injected into the right PBN of wild type mice.

To pharmacologically block the innervation of Sp5C^Tac1^ neurons in PBN, AAV2/9‐hEF1a‐DIO‐hM4D(Gi)‐mCherry‐ER2‐WPRE‐pA virus (4.00 × 10^12^) was injected into the right Sp5C of *Tac1‐Cre* mice. AAV2/9‐hEF1a‐DIO‐EYFP‐WPRE‐pA virus (3.95 × 10^12^) was injected into the right Sp5C as the control. Cannulas (0.41 mm in diameter, RWD Life Science) were implanted into the right PBN. The cannula was secured to the skull with dental cement (coordinate: AP: ‐1.68 mm, ML: ‐5.40 mm, DV: ‐3.05 mm).

400 nL of virus was injected per site. Except for the following experiments, 100 nL was injected per site: CRISPR‐Cas9 mediated *Tac1* gene knock‐down, retrograde labeling (using scAAV2/2Retro‐hSyn‐Cre‐pA and AAV2/2‐Retro‐plus‐hSyn‐FLEX‐tdTomato‐WPRE‐pA), and rabies virus tracing (including helper and rabies virus injection).

### 4.4 Behavioral Tests

During all behavioral tests, mice were head‐fixed using a customized restraint system (Figure S1, China patent application No. 202423024833X). Prior to test, mice were acclimated for 30 min. All behavioral assessments were performed by investigators blinded to treatment group assignments.

### 4.5 Spontaneous Response Test

Spontaneous pain‐related facial expression was assessed using a modified mouse grimace scale (MGS), adapted from the method originally described by Langford et al. [34]. Before stimulation tests at each experimental time point, mice were placed in a customized head‐fixation apparatus to minimize head movement while allowing free body posture. After a 30‐min habituation period, spontaneous facial expression was recorded for 10 min under resting conditions using a high‐resolution camera operating at 25 frames per second (fps). The analyst responsible for evaluating the facial expression videos was blinded to the experimental group assignments.

To avoid potential artifacts caused by initial struggling or adjustment to head fixation, video analysis began 10 s after recording onset. A total of 60 still frames per recording were then systematically extracted at 10‐s intervals (0.1 fps) using FFmpeg software (version N‐113784, FFmpeg Developers).

We chose five predefined orofacial regions of interest (ROIs): ears, eyes, cheeks, whiskers, and nose, in accordance with the established MGS protocol. Each ROI in each frame was assessed using a 3‐point scale (0 = absence of pain‐related alteration, 1 = moderate alteration, 2 = pronounced alteration), based on deviations from the mouse's baseline facial configuration under non‐pain conditions, as previously delineated [34]. Mean regional scores across the 10‐min recording session were calculated and visualized using radar plots. The final MGS score for each mouse was calculated as the average of all its ROI scores. MGS scores were systematically calculated for both baseline and CION‐D14 time points in both sham and CION groups.

Alternatively, spontaneous pain‐related behavior was assessed by quantifying the number of wiping events. Mice were video‐recorded under resting conditions for 10 min before and after stimulation (with a 4‐min recording duration in opto‐activation experiments). Wiping behavior was defined as rapid, directed forelimb movements targeting the affected orofacial region. The total number of wiping events was manually quantified from video recordings during the observation period.

### 4.6 Corn Oil‐Induced Orofacial Grooming Assay

Oil‐induced orofacial grooming behavior was assessed in PBN‐projecting Sp5C *Tac1* gene knockout mice under both head‐fixed and freely moving conditions. To evoke grooming behavior, 100 µL of corn oil was gently applied to the whisker pad using a 1 mL syringe (without needle). Immediately after application, mouse behavior was video‐recorded for 30 min.

For head‐fixed experiments, mice were restrained using a custom head‐fixation apparatus that permitted forelimb movement. Under this condition, grooming behavior did not occur as continuous, stereotyped grooming sequences. Therefore, grooming responses were quantified by counting discrete wiping events, defined as rapid forelimb movements directed toward the whisker pad. The total number of wiping events during the 30‐min recording period was used for analysis.

For freely moving experiments, mice were placed individually in an open recording chamber after corn oil application. In this condition, grooming behavior occurred as continuous grooming bouts. Orofacial grooming was quantified as grooming duration, defined as the cumulative time spent performing orofacial grooming behaviors during the 30‐min recording period. To assess the temporal dynamics of grooming behavior, grooming duration was additionally quantified in consecutive 5‐min time bins across the 30‐min recording session.

### 4.7 Orofacial Mechanical Pain Test with Von Frey Filaments

Orofacial mechanical pain was measured using calibrated von Frey filaments (Touch Test monofilaments kit, North Coast Medical, USA) with a range of forces from 0.008 to 1.0 g. Following 30 min of acclimation, filaments were applied perpendicularly to the right vibrissal pad (V2 trigeminal innervation territory) for 1 s per stimulus. A positive response was defined as an abrupt forepaw wiping motion directed toward the stimulated area. Each filament was tested five times in ascending order (lowest force first) with 10‐s inter‐stimulus intervals. The withdrawal threshold was calculated as the force eliciting ≥ 3 positive responses out of 5 applications, consistent with established sensory hypersensitivity quantification in CION neuropathic pain models.

### 4.8 Orofacial Thermal Sensitivity Test

Orofacial heat sensitivity was assessed using a focused infrared (IR) ray directed at the vibrissal pad. Following Hargreaves’ test principles [78], an IR spot (35–45 mW/cm^2^ intensity) was delivered at 10–12 cm distance. Intensity was calibrated per mouse line/age cohort and held constant within experiments. Cut‐off time was set at 20 s to prevent tissue damage. A positive response was defined as: rapid forepaw wiping directed at the stimulation site or sudden vigorous struggle behavior.

For the innocuous heat (IH) test, a 1.5 mm diameter IR spot was applied from a 12 cm distance to the vibrissal pad. Mice typically show no withdrawal response to IH stimulation during the baseline session. For the noxious heat (NH) test, a 0.8 mm diameter IR spot was applied from a 10 cm distance. For the NH test, IR intensity was adjusted until the mouse withdrawal latency of 10–15 s at the baseline state.

### 4.9 Whole‐Body Plethysmography for Respiratory Rate Measurement

Respiratory rate was measured using whole‐body plethysmography (WBP) (DSI, USA) to assess whether inhibition of PBN^Tac1^ neurons affects basal respiration. Mice were tested at baseline and at day 14 post‐CION surgery (CION‐D14). For each time point, respiratory activity was recorded before and after i.p. injection of deschloroclozapine (DCZ, Ambeed,1 µg/kg).

Prior to recording, mice were placed individually into the plethysmography chamber and allowed to acclimate for 2 days (30 min/day). Respiratory signals on experimental days were recorded for 10 min for analysis. For post‐DCZ measurements, recordings started 30 min after DCZ administration when the drug had taken effect.

Respiratory rate was calculated from the plethysmography signal as breaths per minute and averaged across the 10‐min recording period for each mouse. All data were analyzed using the same parameters across experimental conditions.

### 4.10 Fiber Photometry Recording and Analysis

Calcium signals (Ca^2+^) from PBN^Tac1^ neurons or Sp5C neuron axons in PBN were recorded via fiber photometry with optical fibers. Head‐fixed mice were acclimated for 30 min pre‐recording.

To test neuronal activity in response to mechanical stimulation, mouse vibrissal pad received innocuous (0.02 g) and noxious (0.4 g) mechanical stimuli sequentially, with Ca^2+^ signals recorded for 10 s post‐stimulation. For neuronal activity responding to thermal IH and NH stimuli (using heat test protocols), Ca^2+^ signals were recorded for 20 s per trial. 3 trials responses were collected per stimulus type.

Generally, Ca^2+^ signals were recorded pre‐surgery and on day 14 post‐surgery in CION mice. Ca^2+^ signals in sham controls were recorded at identical timepoints.

At experiment termination, all animals were perfused and brain tissues were collected for histological examination of optical fiber location and virus expression. Data enrollment was based on the correct optical fiber placement and virus expression. Signal processing was performed by MATLAB (R2017a), with stimulus onset marked by real‐time analog input synchronization. After subtracting photodetector noise, data were smoothened using a 20‐ms moving average filter. Calcium transient changes (ΔF/F) from ‐5 s to 20 s relative to stimulus onset (0 s) were calculated as (F‐F_0_)/F_0_, where F_0_ represents the mean fluorescence during the pre‐stimulus baseline period (‐5 s to 0 s). For mechanical stimulation experiments, calcium transient changes from ‐5 s to 10 s relative to stimulus onset were analyzed.

All fiber photometry recordings were initially analyzed at the trial level. In experiments recording PBN^Tac1^ neuronal soma activity, calcium responses were robust and highly reproducible across trials within individual mice. Therefore, trial‐level responses were averaged within each mouse to generate a single representative value per condition, and each mouse contributed a single data point to group‐level analyses and graphical representations.

In contrast, in experiments recording Sp5C axonal calcium signals in the PBN, stimulation‐evoked responses were weaker and exhibited greater trial‐to‐trial variability. To better visualize stimulus‐evoked calcium responses under these conditions, individual trial responses were plotted as data points in group comparisons. Each plotted data point represents a single trial, with 3 trials from each animal.

### 4.11 In Vivo Optogenetic Manipulation

An optical fiber was implanted into the ventral part of PBN at least 1 week before behavioral tests.

Optogenetic stimulation (473‐nm blue light, 5‐ms pulses) followed by a 4 min protocol: 30 s off / 30 s on cycles repeated 4 times. Pre‐test verification confirmed 1 mW fiber‐tip irradiance and 5 Hz stimulation frequency (5 ms on, 195 ms off; 5 pulses/s) ‐ parameters eliciting robust pain‐like wiping behavior. The stimulation condition was applied in wiping behavior quantification and orofacial mechanical threshold test.

During the wiping behavior test, freely moving mice were filmed with bilateral cameras. For the orofacial threshold test during laser stimulation, mice were gently restrained in tester's hand with a soft towel during filaments application.

### 4.12 Real Time Place Avoidance Test

Mice underwent real time conditioned place avoidance test in a two‐chamber apparatus (black‐walled vs. white‐walled) for 45 min. Each mouse was tested once to prevent memory confounds. After a 15‐min pre‐conditioning baseline, 470‐nm light stimulation (10 Hz, 5‐ms pulses, 1 mW fiber‐tip irradiance) was delivered to the right PBN whenever the mice entered or stayed in the black chamber during 15‐min conditioning phase. Stimulation ceased upon chamber exit. This was followed by a 15‐min post‐conditioning phase with no stimulation. Time spent in the black chamber was quantified for 3 phases.

### 4.13 RNAscope In Situ Hybridization

RNAscope multiplex fluorescent in situ hybridization (ISH, RNAscope Multiplex Fluorescent Detection Kit v2, ACDBio) was performed to: (1) characterize *Tac1/Tacr1/Sst* expression in the PBN of sham vs. 14‐day CION mice; (2) quantify *Tac1*‐positive proportions in PBN‐projecting Sp5C and Sp5C^Tac1^ neurons; and (3) assess *Tac1* knockdown efficiency in Sp5C. Brain samples were collected after transcardiac perfusion with ice‐cold saline followed by 4% paraformaldehyde (PFA) under anesthesia, then post‐fixed overnight in 4% PFA at 4°C. Given cryoprotection in 30% sucrose (4°C till tissue sank), samples were embedded in OCT (Sakura Finetek), frozen on dry ice for 1 h and sectioned at 20 µm using a cryostat (Leica Microsystems). Sections were mounted on adhesive microscope slides (Citotest), air dried overnight (RT), and stored at ‐80°C until use.

For hybridization, slides were washed in ddH_2_O (10 s), baked (60°C, 30 min), post‐fixed (4% PFA, 4°C, 15 min), dehydrated in graded ethanol (50%, 70%, 100%, 5 min each, RT), and treated with hydrogen peroxide (10 min, RT). Slides were then transferred to a boiling retrieval reagent (5 min) and rinsed in ddH_2_O (twice, RT). Protease III digestion (40°C, 30 min) preceded hybridization with targeted probes (*c‐Fos*, *Tacr1*, *Tac1*, *Sst*, and *mCherry;* see Table 1) in HybEZ II Oven (Advanced Cell Diagnostics, Inc.). Signal amplification followed manufacturer protocols. After final washes, sections were counterstained with DAPI and mounted with coverslips. Imaging was performed using a confocal microscopy (Zeiss LSM900 or Evident FV3000). Given that the *Tac1* is predominantly expressed in the superficial layers of the Sp5C, the boundaries of laminae I‐II were determined based on the distribution of the *Tac1*‐positive neurons.

**TABLE 1:** Materials used in this study.

REAGENT or RESOURCE	SOURCE	IDENTIFIER
Antibodies		
Rabbit anti‐GFP	Invitrogen	Cat#A11122; RRID: AB_221569
Rabbit anti‐DsRed	Clontech	Cat#632496; RRID: AB_10013483
DAPI	Beyotime	Cat#C1002; RRID: AB_3675433
Goat‐anti‐CGRP	Abcam	Cat#ab36001; RRID: AB_725807
Chicken anti‐Neurofilament heavy polypeptide (NF200)	Abcam	Cat#ab4680; RRID: AB_304560
Guinea pig anti‐SCN11A	Alomone	Cat#ASC‐017‐GP; RRID: AB_2340966
Doneky anti‐Goat IgG Alexa Fluor 488	Invitrogen	Cat#A32814; RRID: AB_2762838
Donkey anti‐Chicken IgM ‐Cy3	Jackson ImmunoResearch Laboratories	Cat#703‐165‐155; RRID: AB_2340363
Donkey anti‐Guinea Pig IgG Alexa Fluor 647	Jackson ImmunoResearch Laboratories	Cat#706‐605‐148; RRID: AB_2340476
Donkey anti‐Rabbit IgG Alexa Fluor 488	Jackson ImmunoResearch Laboratories	Cat#711‐545‐152; RRID: AB_2313584
Donkey anti‐Rabbit IgG Cy3	Jackson ImmunoResearch Laboratories	Cat#711‐165‐152; RRID: AB_2307443
Virus strains		
AAV2/9‐hEF1a‐DIO‐GCaMP6s‐WPRE‐pA	Taitool	Cat#S0351‐9
AAV2/8‐hSyn‐DIO‐EGFP‐WPRE‐pA	Taitool	Cat#S0746‐8
AAV2/9‐hSyn‐DIO‐hM4D(Gi)‐eGFP‐WPRE‐pA	Taitool	Cat#S0286‐9
AAV2/8‐hSyn‐EGFP‐WPRE‐pA	Taitool	Cat#S0237‐8
AAV2/9‐hSyn‐EGFP.PreSynapse‐WPRE‐pA	Taitool	Cat#0789‐9
AAV2/8‐hSyn‐HA‐hM4D (Gi)‐mCherry‐WPRE	OBIO	Cat#TS0215
AAV1‐FLEX‐split TVA‐P2A‐EGFP‐P2A‐tTA	Lavin et al. [75]	N/A
AAV‐TREtight‐mTagBFP2‐N2cG	Zhang et al. [76]	N/A
CSV‐N2△G‐4mCherry (EnvA)	Zhang et al. [77]	N/A
AAV2/9‐hSyn‐hChR2(H134R)‐mCherry‐WPRE‐pA	Taitool	Cat#S0165‐9
AAV2/9‐hEF1a‐DIO‐EYFP‐WPRE‐pA	Taitool	Cat#S0196‐9
AAV2/9‐hEF1a‐DIO‐hM4D(Gi)‐mCherry‐ER2‐WPRE‐pA	Taitool	Cat#S0863‐9
scAAV2/2Retro‐hSyn‐Cre‐pA	Taitool	Cat#S0292‐2R
AAV2/9‐hEF1a‐DIO‐hChR2(H134R)‐EYFP‐WPRE‐pA	Taitool	Cat#S0199‐9
AAV2/9‐hSyn‐GCaMP6s‐WPRE‐pA	Taitool	Cat#S0225‐9
AAV2/2 Retro plus‐hSyn‐FLEX‐tdTomato‐WPRE‐pA	Taitool	Cat#S0255‐2RP
AAV2/9‐hSyn‐DIO‐mCherry‐pA	Taitool	Cat#S0240‐9
AAV2/2Retro‐hSyn‐mCherry‐3Flag‐WPRE‐pA	Taitool	Cat#S0238‐2R
PAAV‐U6‐sgCtrl‐CAG‐FLEX‐mCherry‐WPRE (AAV2/8) PSE7606	This study	Cat#E591
PAAV‐U6‐sgTac1‐3‐U6‐sgTac1‐4‐CAG‐FLEX‐mCherry (AAV2/8) PSE7607	This study	Cat#G113
AAV2/9‐hSyn‐hChR2(H134R)‐EYFP‐WPRE‐pA	Taitool	Cat#S0318‐9
AAV2/9‐hEF1a‐fDIO‐hM4D(Gi)‐mCherry‐ER2‐WPRE‐pA	Taitool	Cat#S0336‐9
scAAV2/1‐hSyn‐FLPo‐pA	Taitool	Cat#S0294‐1
AAV2/9‐hsyn‐Con/Fon‐hM3D(Gq)‐mCherry‐WPRE‐pA	Taitool	Cat#S0684‐9
AAV2/9‐hSyn‐Con/Fon‐EYFP	Taitool	Cat#S0267‐9
AAV2/2Retro‐hSyn‐EGFP(‐3Flag)‐WPRE‐pA	Taitool	Cat#S0237‐2R
Chemicals		
Normal donkey serum	Jackson ImmunoResearch Laboratories	Cat#017‐000‐121; RRID: AB_2337258
Fish skin gelatin	Sigma‐Aldrich	Cat#G7041
Trizol	Invitrogen	Cat#15596026CN
TTX	Taizhou Kangte Biotechnology Co.	Cat#201206
4‐AP	Tocris	Cat#0940
NBQX	Sigma‐Aldrich	Cat#N183
D‐AP5	Tocris	Cat#0106
Picrotoxin	Tocris	Cat#1128
Avidin conjugated Alexa Fluor 647	ThermoFisher Scientific	Cat#S21374
CTB‐488	Invitrogen	Cat#C34775
CTB‐555	Invitrogen	Cat#C34776
435/455 blue fluorescent Nissl stain	Invitrogen	Cat#N21479
IB4 conjugated Alexa Fluor 647	Invitrogen	Cat#I32450
Critical commercial assays		
RNAscope Multiplex Fluorescent Reagent Kit v2	Advanced Cell Diagnostics, Inc.	Cat#323270
RNAscopeTM Probe‐Mm‐Tac1	Advanced Cell Diagnostics, Inc.	Cat#410351
RNAscopeTM Probe‐Mm‐c‐Fos	Advanced Cell Diagnostics, Inc.	Cat#316921
RNAscopeTM Probe‐Mm‐Tacr1	Advanced Cell Diagnostics, Inc.	Cat#428781
RNAscopeTM Probe‐Mm‐Sst	Advanced Cell Diagnostics, Inc.	Cat#404631
RNAscopeTM Probe‐Mm‐mCherry	Advanced Cell Diagnostics, Inc.	Cat#431201
Other		
Fiber photometry system	Thinker Tech Nanjing Bioscience Inc (China)	N/A
Whole‐body plethysmography	Data Science International	Buxco FinePointe^TM^
Olympus confocal microscope	Olympus	FV3000
Carl Zeiss confocal microscope	Carl Zeiss	LSM900
Cannula	RWD (China)	Cat#62003
Optical fiber	Inper	Cat#554L3
Infrared ray intensity test meter	Sanpometer (China)	IR850‐940

### 4.14 Immunofluorescent Staining

Brain and/or TG were collected as described in RNAscope ISH section. Then tissues were embedded in OCT (Sakura Finetek) and cryosectioned at 15–30 µm using a cryostat (Leica Microsystems).

For brain immunochemistry staining, sections were washed with PBS (5 min), blocked in 3% normal donkey serum (Jackson ImmunoResearch) / 3% Triton X‐100 (Sigma) / PBS (30‐60 min, RT) and incubated in primary antibodies (4°C, overnight). After PBS washes (3 × 10 min), sections were incubated with secondary antibodies and DAPI (1:10,000, 2 h, RT). Following final PBS washes (3 × 10 min), slides were mounted with 70% glycerol in PBS. Please see Table 1 for detailed information about primary and secondary antibodies.

To check the expression of GFP‐tag virus, primary rabbit anti‐GFP antibody (1:500) and secondary Alexa Fluor 488‐conjugated donkey anti‐rabbit IgG antibody (1:200) were used. To check the expression pattern of mCherry or tdTomato tag virus, primary rabbit anti‐DsRed antibody (1:500) and secondary donkey anti‐rabbit Cy3 antibody (1:500) were used.

For TG immunochemistry staining, mounted sections were PBS‐washed (5 min), blocked in 0.5% fish skin gelatin (Sigma) / 3% Triton X‐100 (Sigma) / PBS (30‐60 min, RT), and incubated with primary antibodies (4°C, overnight). After PBS washes (3 × 10 min), secondary antibodies were applied (2 h, RT). After another PBS washes (3 × 10 min) to remove secondary antibodies, sections were then counterstained with DAPI (1:10,000) or Nissl (1:200) (20 min, RT), washed, and mounted.

To stain for TG neurons, primary goat anti‐CGRP antibody (1:3,000), chicken anti‐Neurofilament heavy polypeptide (NF200) antibody (1:3,000), guinea pig anti‐SCN11A antibody (1:200). Secondary donkey anti‐goat IgG Alexa Fluor 488‐conjugated antibody (1:500), donkey anti‐chicken IgM ‐Cy3 antibody (1:500) and donkey anti‐guinea pig IgG Alexa Fluor 647 antibody (1:500) with IB4 Alexa Fluor 647 conjugate (1:500) were used, respectively.

To stain recorded neurons in ex vivo electrophysiology, post‐recorded slices were post‐fixed in 4% PFA (≥ 24 h, 4°C), PBS‐washed (3 × 10 min), block as mentioned, and incubated with Avidin‐Alexa Fluor 647 (1:500, 2 h, RT), then washed and mounted in 70% glycerol in PBS.

### 4.15 Image Acquisition and Analysis

Brain and TG sections were imaged using a VS200 slide scanner (Olympus). Anatomical delimitations of PBN, Sp5C, and other regions were determined using the mouse brain atlas [79]. To verify the accuracy of the viral injection site, serial coronal sections (≥ 3 sections) encompassing the target brain region were examined, and the extent of viral spread was visualized and mapped. Cell quantification utilized ≥ 3 coronal sections per brain area of one mouse, manually counted via ImageJ's Cell Counter plugin (NIH). For analysis, PBN^Tac1^ upstream projections were mapped and counted in the sixth‐level brain regions, while relative *Tac1* mRNA levels were quantified by fluorescence intensity (ImageJ). Data were represented as mean ± standard error of the mean (mean ± SEM) with statistical analyses performed using GraphPad Prism 9 and specific tests detailed per experiment.

### 4.16 Chemogenetic Manipulation

Clozapine N‐oxide (CNO, Tocris) was dissolved in dimethyl sulfoxide (DMSO, Ambeed) to prepare a 10 mg/mL stock solution. For systemic chemogenetic inhibition, CNO was diluted in sterile saline and i.p. administered at 3 mg/kg on test days. For testing orofacial pain‐like phenotypes following the chemogenetic activation of TG‐Sp5C^Tac1^ pathways, different concentrations of CNO (0.1, 0.5, 1, or 3 mg/kg) were i.p. injected.

For chemogenetic inhibition of the axons of PBN‐projecting Sp5C^Tac1^ neurons, 5 µg of CNO was infused over 5 min via a PBN implanted cannula using a microinfusion pump (0.1 µL/min flow rate).

To assess potential effects of PBN^Tac1^ neuron inhibition on basal respiratory rate, 1 µg/kg of DCZ was used for systemic chemogenetic inhibition of hM4Di‐expressing PBN^Tac1^ neurons. DCZ was initially dissolved in DMSO to prepare a 1 mg/mL stock solution. On test days, the DCZ stock was diluted in sterile saline (1:99, v/v) to obtain the working solution.

### 4.17 Ex Vivo Electrophysiology

Slice electrophysiology was conducted as described previously [72]. Briefly, mice were anesthetized with isoflurane (RWD Life Science) and perfused with ice‐cold N‐methyl‐d‐glucamine (NMDG) based cutting solution containing (in mM): 93 NMDG, 93 HCl, 30 NaHCO_3_, 20 HEPES, 2.5 KCl, 1.2 NaH_2_PO_4_, 10 MgCl_2_, 0.5 CaCl_2_, 25 glucose, 3 myo‐inositol, 1 sodium‐L‐ascorbate, 5 ethyl pyruvate (∼305 mOsm). Brain was rapidly dissected and adhered to a cutting platform via a tissue glue (PATTEX). Coronal sections were prepared: three 250 µm sections for PBN and four 230 µm sections for Sp5C using a vibratome (Leica Microsystems). Slices were in turn transferred to artificial cerebral spinal fluid (ACSF) containing (in mm): 126 NaCl, 2.5 KCl, 1.25 NaH_2_PO_4_, 2 MgCl_2_, 2 CaCl_2_, 26 NaHCO_3_, and 10 glucose (∼300 mOsm) at 34°C for 30 min before being passively equilibrated to room temperature for at least 1 h before recording. All solutions were bubbled with 95% O_2_/5% CO_2_ for at least 30 min prior to use. Brian slices were then transferred to the recording chamber, which was continuously perfused with ACSF at the rate of 2 mL/min maintained at 30°C by an automatic temperature controller (TC‐324C, Warner Instruments). Fluorescence labeled neurons or fibers were visualized with 40× objective lens on a BX51WI microscope (Olympus) using LED illumination (FluoCa). Patch electrodes (4‐6 mΩ) were pulled using a two‐stage puller (Narishige), and filled with cesium‐based intracellular solution containing the following in gradients (in mm): 130 CsMeSO_3_, 1 MgCl_2_, 1 CaCl_2_, 10 HEPES, 2 QX‐314, 11 EGTA, 2 Mg‐ATP, 0.3 Na‐GTP (pH 7.3, 290 mOsm) during the recording. Biocytin (2 mg/mL) was included in the intracellular solutions for post‐hoc recovery of recorded neurons. All chemicals were purchased from Sigma–Aldrich.

Data were acquired using a Multiclamp 700B amplifier (Molecular Devices) and Clampex software (pClamp10, Molecular Devices). Signals were low‐pass filtered at 10 kHz and digitized at 10 kHz (Digidata 1550B). Resting membrane potentials (RMP) of neurons were recorded shortly after break‐in. Neurons exhibiting significant leak current were discarded without further recording. Series resistance (∼14–18 MΩ) was monitored throughout the recording. Neurons were excluded from the analysis if the change of series resistance exceeded 20% the original value. For optogenetic stimulation experiment, ChR2 was activated using an LED illumination system (λ = 473 nm, 1 ms, 10 mW), which was controlled by pClamp 10 software. Blue‐light‐evoked EPSCs and IPSCs were recorded while clamping the membrane potential at −70 mV and 0 mV, respectively. To test whether the recorded IPSCs were mediated by the GABA receptor, 50 µm picrotoxin was added to ACSF. To test whether the recorded inward currents were excitatory currents, 10 µm NBQX and AP5 were added to ACSF. To test whether the postsynaptic currents recorded were monosynaptic connections, 5 µm tetrodotoxin (TTX) and 1 mm 4‐aminopyridine (4‐AP) were added to ACSF.

Stock solutions of drugs were made and stored at ‐20°C and further diluted to their final concentration in ACSF on the day of the recording. We used a Python‐based pyABF and Electrophys Feature Extraction Library (EFEL) package or Clampfit 10.7 s (Molecular Devices) for data visualization and analysis.

### 4.18 Generation of Smart‐seq3 Libraries and Sequencing of Single PBN‐Projecting Sp5C Neurons

Generation of Smart‐seq3 libraries was conducted according to Smart‐seq3 protocol [80]. Superficial layers of Sp5C were collected and incubated in a choline chloride solution containing 20 U/mL papain (Worthington) and 100 U/mL DNase I (Worthington) for 15 min at 37°C, and this was followed by digestion with 1 mg/mL protease (Sigma) and dispase (Worthington) for 20 min at 25°C. Next, the post‐digested tissues were gently triturated using fire‐polished glass Pasteur pipettes to obtain single‐cell suspensions. The single‐cell suspensions were then centrifuged at 400 g, at 4°C for 5 min, and the supernatant was discarded. The cells were re‐suspended with choline chloride solution containing 2% FBS (MACS). The choline chloride solution contained (in mM): 92 choline chloride, 2.5 KCl, 1.2 NaH_2_PO_4_, 30 NaHCO_3_, 20 HEPES, 25 glucose, 5 sodium ascorbate, 2 thiourea, 3 sodium pyruvate, 10 MgSO_4_·7H_2_O, 0.5 CaCl_2_·2H_2_O, and 12 N‐acetyl‐L‐cysteine. Fluorescence labeled cells were manually selected under a microscope and the cytosol of each single cell was transferred individually to a tube with lysis buffer, containing 5% Poly‐ethylene Glycol 8000 (Sigma), 0.1% Triton X‐100 (Sigma), 0.5 U/µL RNase Inhibitor (Takara), 0.5 m OligodT30VN (5′‐biotin‐ACGAGCATCAGCAGCATACGATTTTTTTTTTTTTTTTTTTTTTTT TTTTTTVN; Sangon), 0.5 mm dNTPs (Thermo Scientific). All procedures were completed within 2.5 h after the animals were euthanized. To facilitate cell lysis and denaturation of the RNA, plates were incubated at 72°C for 10 min and immediately placed on ice afterward. Next, 1 µL of reverse transcription mix was added per sample, containing 25 mm Tris‐HCL, pH 8.3 (Sigma), 30 mm NaCl (Ambion), 1 mm GTP (Thermo Scientific), 2.5 mm MgCl_2_ (Ambion), 8 mm DTT (Thermo Scientific), 0.5 U/µL RNase Inhibitor (Takara), 2 µm of different Smart‐seq3 TSOs (5′‐biotin‐AGAGACAGATTGCGCAATGNNNNNNNNrGrGrG‐3′; Sangon) and 2 U/µL of Maxima H‐minus reverse transcriptase enzyme (Thermo Scientific). Reverse transcription and template switching were carried out at 42°C for 90 min, followed by 10 cycles of 50°C for 2 min and 42°C for 2 min. The reaction was terminated by incubating at 85°C for 5 min. PCR pre‐amplification was performed directly after reverse transcription by adding 6 µL of PCR mix, to achieve final concentration of: 1× KAPA HiFi PCR buffer (containing 2 mm MgCl_2_ at 1× (Roche), 0.02 U/µL DNA polymerase (Roche), 0.3 mm dNTPs, 0.5 µm Smartseq3 forward PCR primer (5′‐TCGTCGGCAGCGTCAGATGTGTATAAGAGACAGATTGCGCAATG‐3′; Sangon) and 0.1 µm Smartseq3 reverse PCR primer (5′‐ACGAGCATCAGCAGCATACGA‐3′; Sangon). PCR was cycled as follows: 3 min at 98°C for initial denaturation, 19 cycles of 20 s at 98°C, 30 s at 65°C, and 4 min at 72°C. Final elongation was performed for 5 min at 72°C. DNA library were built followed TruePrep DNA Library Prep Kit V2 for Illumina TD503 (Vazyme). Libraries were pooled and sequenced on the Illumina Novaseq platform, with an average read count of approximately 20 million paired end reads per sample.

### 4.19 Single‐Neuron RNA Sequencing Data Analysis for Smart‐seq3 Libraries

Single‐neuron RNA‐seq raw data were pre‐processed and analyzed. Briefly, initial quality assessment of concatenated replicate raw sequencing reads from each library was conducted using FastQC [81] (Version 0.11.9) to ensure minimal PCR duplication and sequencing quality. Reads were aligned to the hg38 or mouse mm10 genome using HISAT2 [82] (Version 2.2.1). Multiple‐aligned reads were discarded, and remaining transcript reads were counted using featureCounts [83] (Version 2.1.1). Expression levels were normalized to Transcripts Per Million (TPM) calculated as the following Equation (1):

(1) $$\begin{array}{ccc} & & TPM\\ & & =10^{6}*\frac{reads\;mapped\;to\;transcript/transcript\;length}{Sum\;\left(reads\;mapped\;to\;transcript\;/transcript\;length\right)} \end{array}$$

Differential gene expression analysis was performed using the DESeq2(v1.48.1) [84], excluding genes detected in fewer than two cells. Adjusted *p* value (i.e., false discovery rate, FDR) < 0.05 and Log_2_ (Fold Change) > 1 or Log_2_ (Fold Change) < ‐1 was set as the statistical threshold for significant DEGs, which were plotted as a volcano graph and a heatmap. The key DEGs were integrated into the STRING database to derive their protein–protein interaction (PPI) network [85]. We established the screening parameters for the organism as “Mus musculus”. On the basis of PPI network, Gene Ontology (GO) pathway analysis and Kyoto Encyclopedia of Genes and Genomes (KEGG) pathway enrichment analysis of DEGs in modules were performed. GO enrichment analysis includes Biological Process (BP), Molecular Function (MF), and Cellular Component (CC) analysis.

### 4.20 Statistical Analyses

Data are presented as mean ± SEM. Normality was assessed for all datasets before conducting statistical analysis. Detailed statistical tests and post hoc analyses employed in each experiment were performed using GraphPad Prism 9 (GraphPad Software).

## Author Contributions

J.D., L.S., and J.‐J.W. designed the research. L.S., J.‐J.W., X.‐Y. Li, J. Li, Q.‐T.Y., and Z.‐H.W. performed the experiments. W.‐K.L. and T.‐L.C. designed the gRNAs. X.‐P.G. and L.J. provided the RV virus. L.S., J.‐J.W., X.‐Y.Li, and X.‐Y.Lin analyzed the data and generated the figures. J.D. and L.S. wrote the manuscript.

## Funding

This project is supported by funding as follows: National Science and Technology Innovation 2030 Major Program (2022ZD0207300) and National Natural Science Foundation of China (31825013, 82101320, and 82222020).

## Conflicts of Interest

The customized head‐fixation system shown in Figure S1 was from a patent application (202423024833X, China) authored by J.D., L.S., and J.‐J.W.

## Supporting information




**Supporting File**: advs74763‐sup‐0001‐SuppMat.docx.

## Data Availability

Single‐neuron sequencing datasets generated in the current study are available in the GSE322600. For further information and requests regarding resources and reagents, please contact the lead contact, Juan Deng (juandeng@fudan.edu.cn), who will fulfill these requests.
