# Supplementary material for: A Tac1‐Expressing Brainstem Pathway Underlies the Pathogenesis of Trigeminal Neuralgia
Source: Adv Sci (Weinh). 2026 Mar 13;13(29):e16310. doi: 10.1002/advs.202516310 (PMC13205611; doi:10.1002/advs.202516310)
Supplement: Supplementary file 1 — Supporting File: advs74763‐sup‐0001‐SuppMat.docx. [file ADVS-13-e16310-s001.docx]

Supporting information for

**A *Tac1*-expressing brainstem pathway underlies the pathogenesis of trigeminal neuralgia**

Liting Sun^1^†*, Jia-Jia Wang^1^†, Xiang-Yu Li^1^†, Xin-Yi Lin^1^, Juan Li^1^, Qiu-Tong Yu^1^, Zi-Han Wang^1^, Xue-Ping Gao^2^, Lei Jin^2^, Wei-Ke Li^1^, Tian-Lin Cheng^1^, Juan Deng^1,3^*

^1^Department of Anesthesiology, Huadong Hospital, State Key Laboratory of Medical Neurobiology, Institute for Translational Brain Research, MOE Frontiers Center for Brain Science, Fudan University, Shanghai 200032, China

^2^Lingang Laboratory, Shanghai 200031, China

^3^Lead contact

† These authors contributed equally

*Correspondence: juandeng@fudan.edu.cn and litingsun@fudan.edu.cn

**This PDF file includes:**

Figs. S1 to S9

Supplementary Materials

**Supplementary Figures and captions**

**
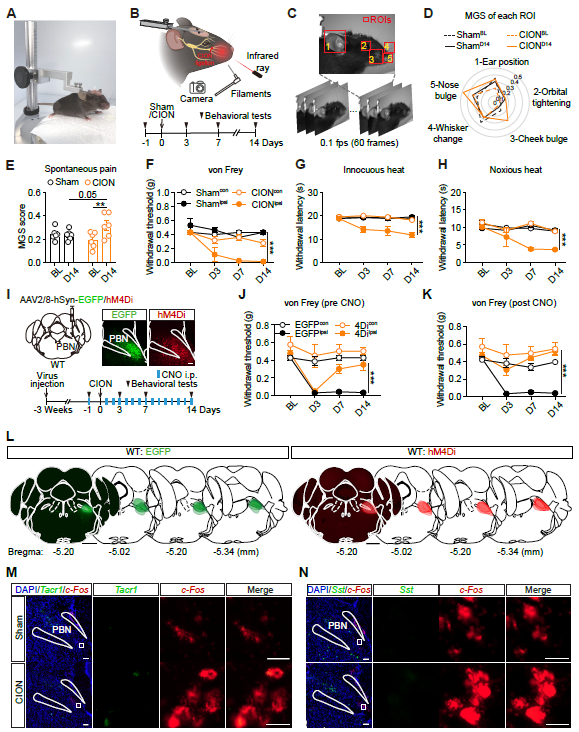
**

**Fig. S1, related to Fig. 1. PBN is activated in a mouse model of trigeminal neuropathic pain.** **(A),** The customized head-fixation system for mice. **(B),** A schematic and timeline for behavioral tests before and after Sham/CION surgery. **(C),** Analysis strategy for spontaneous response using the modified mouse grimace scale (MGS) score. 60 still frames (0.1 fps) were extracted from a 10-min session, and each frame was scored based on alteration in five predefined orofacial regions of interest (ROIs): ears, eyes, cheeks, whiskers, and nose. Each ROI was assigned a score from 0 to 2 (3-point scale: 0, 1 or 2), reflecting increasing degrees of pain-related facial change, with higher scores indicating greater deviation from the non-pain facial appearance. **(D),** A radar map illustrates MGS scores of each ROI, each ROI score were calculated as the mean value of 60 frames across 10-min session, at baseline (BL) and day 14 (D14) after sham or CION surgery. **(E),** MGS scores of the sham and CION group at BL and D14. ***p* < 0.01, by two-way ANOVA (n = 6 for each group). **(F),** Mechanical withdrawal responses evoked by von Frey filaments of mice in two groups at distinct timepoints of BL, D3, D7 and D14. ****p* < 0.001, by two-way ANOVA (n = 6 for each group). **(G),** Thermal withdrawal latency evoked by innocuous heat (IH) measured in mice from two groups at different timepoints. ****p* < 0.001, by two-way ANOVA (n = 6 for each group). **(H),** Thermal withdrawal latency evoked by noxious heat (NH) measured in mice from two groups at different timepoints. ****p* < 0.001, by two-way ANOVA (n = 6 for each group). **(I),** A schematic and timeline for the experiment. The images showing examples of virus expression pattern in EGFP and hM4Di groups. Scale bar: 100 μm. **(J-K),** Mechanical withdrawal responses evoked by von Frey filaments of mice in two groups before and after CNO injection. ****p* < 0.001, by two-way ANOVA (n = 8 for each group). **(L),** EGFP and hM4Di virus expression pattern in PBN of experiment showed in **I**. Scale bar: 1 mm. (**M**), Show case for gene expression pattern of *c-Fos* (red) and *Tacr1* (green) in the right PBN in both groups. Blue: DAPI. Scale bar: 100 μm, inset scale bar: 20 μm. **(N),** Show case for gene expression pattern of *c-Fos* (red) and *Sst* (green) in the right PBN in both groups. Blue: DAPI. Scale bar: 100 μm, inset scale bar: 20 μm.


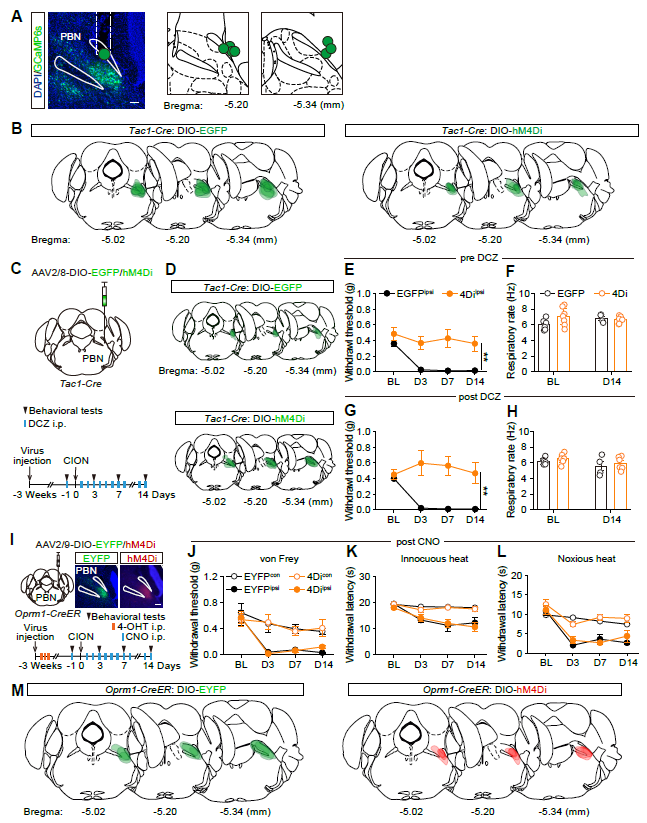


Fig. S2, related to Fig. 1. Inhibition of PBN^Tac1^ neurons attenuates trigeminal neuropathic pain without influence on respiration. (A), The example image and summarized atlases showing the terminal location of optic fibers implanted in PBN for fiber photometry recording. Green dot: optic fiber terminal location (related to Fig.1 D-G). Scale bar: 100 μm. (B), The brain atlases showed the expression pattern of DIO-EGFP and DIO-hM4Di virus in PBN (related to Fig.1 H-O). (C), A schematic and timeline for the respiratory test experiment. (D), The brain atlases showed the expression pattern of DIO-EGFP and DIO-hM4Di virus in PBN of respiratory test experiment. (E, G), Mechanical withdrawal responses evoked by von Frey filaments measured in mice from both groups pre DCZ (E) and post DCZ i.p injection (G). ***p* < 0.01, by two-way ANOVA. (F, H), Respiratory rate of mice measured in mice from both groups pre DCZ (F) and post DCZ i.p injection (H) (EGFP, n = 6 for BL, n = 4 for D14; hM4Di, n = 6). (I) A schematic and timeline for the experiment. The images showing examples of virus expression pattern in EYFP and hM4Di groups of *Oprm-1-CreER* mice. Scale bar: 100 μm. 4-OHT: 4-Hydroxytamoxifen. (J-L), Mechanical withdrawal responses evoked by von Frey filaments (J), and thermal withdrawal latency evoked by innocuous heat (K) and noxious heat (L) measured in mice from both groups (EYFP, n = 5; hM4Di, n = 6). (M), The brain atlases showed EYFP and hM4Di virus expression pattern in PBN of *Oprm1-CreER* mice.


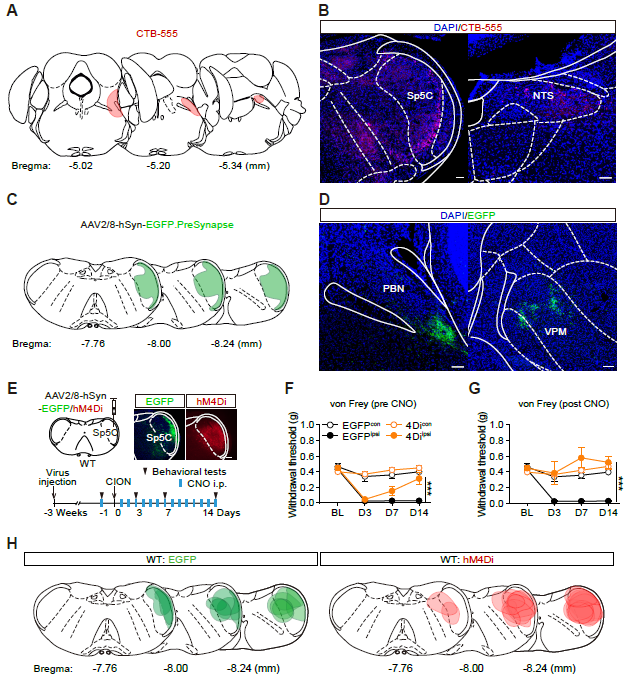


Fig. S3, related to Fig. 2. Sp5C-PBN circuit contributes to trigeminal neuropathic pain. (A), The brain atlases showed the spread range of CTB-555 in PBN. (B), Images showing the distribution pattern of CTB-555-positive neurons in the right Sp5C and the right NTS. Red: CTB-555, blue: DAPI. Scale bar: 100 μm. (C), The brain atlases showed the spread range of EGFP.PreSynapse in Sp5C. (D), Image showing the distribution pattern of EGFP-positive neuronal fibers in the right PBN and the ventral posteromedial nucleus of the thalamus (VPM). Green: EGFP, blue: DAPI. Scale bar: 100 μm. (E), A schematic and timeline for the experiment. The images showing examples of virus expression pattern in EGFP and hM4Di groups. Scale bar: 100 μm. (F-G), Mechanical withdrawal responses evoked by von Frey filaments measured before and after CNO application in mice from both groups. ****p* < 0.001, by two-way ANOVA (EYFP, n = 6; hM4Di, n = 8). (H), The brain atlases showed the expression pattern of EGFP and hM4Di virus in Sp5C for experiment showed in E.


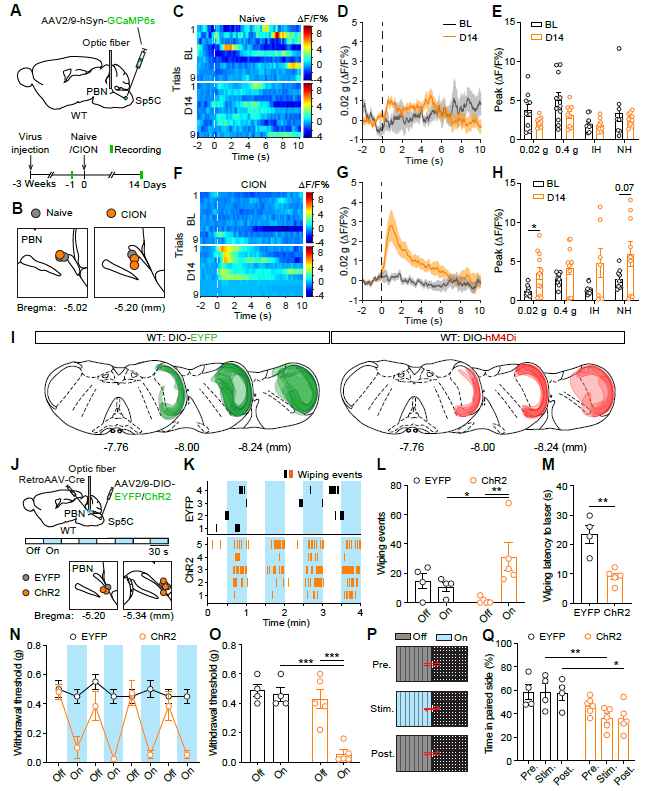


Fig. S4, related to Fig. 2. Sp5C-PBN circuit contributes to trigeminal neuropathic pain. (A), A schematic and timeline for fiber photometry recording of Sp5C-PBN pathway. The images showing examples of GCaMP6s virus expression pattern and optical fiber implantation sites in PBN. Scale bar: 100 μm. (B), The brain atlases showing optical fiber implantation sites in PBN of mice in Naive and CION groups. Gray dot: optic fiber terminals in Naive group, orange dot: optic fiber terminals in CION group. (C-H), Heatmaps showing innocuous 0.02 g von Frey filament-evoked fluorescence changes recorded from mice in Naive (C) and CION (F) groups. Curves showing 0.02 g von Frey filament-evoked fluorescence signals recorded from mice in Naive (D) and CION (G) groups. Dash lines represent onset of stimuli. Peak value of stimulus-evoked fluorescence signals recorded from mice in Naive (E) and CION (H) groups (the dot represents one stimulation-evoked trial). BL: baseline, D14: 14 days after CION surgery. **p* < 0.05, by unpaired t test (n = 9 trials from 3 mice for each group). (I), The brain atlases showed the expression pattern of DIO-EYFP and DIO-hM4Di virus in Sp5C (related to Fig.2 O-V). (J), A schematic for opto-genetic activation of the Sp5C–PBN pathway. The brain atlases showing optical fiber implantation sites in PBN of EYFP and ChR2 groups. (K), Raster plot of wiping behavior observed in mice from EYFP and ChR2 groups. Blue rectangles represent blue laser-on period (n = 4-5 for each group). (L), Wiping events observed in mice from two groups during laser-off and laser-on period. **p* < 0.05, ***p* < 0.01, by two-way ANOVA. (M), Wiping behavior latency detected from mice in both groups during laser-on period. ***p* < 0.01, by unpaired t test. (N), Average mechanical threshold of the right whisker pad detected from mice in both groups during laser-on and laser-off period. (O), Mechanical threshold of the right whisker pad detected from mice in both groups during laser-on and laser-off period. ****p* < 0.001, by two-way ANOVA (EYFP, n = 4; ChR2, n = 5). (P), A schematic for real time place avoidance (RTPA) test of mice during laser-off (Pre. and Post.) and laser-on (Stim.) period paired in the left chamber. Stim.: stimulation. (Q), Percentage of time spent in Stim.-paired side detected from mice in both groups in each stage of RTPA test. *p < 0.05, **p < 0.01, by two-way ANOVA (EYFP, n = 4; ChR2, n = 5).


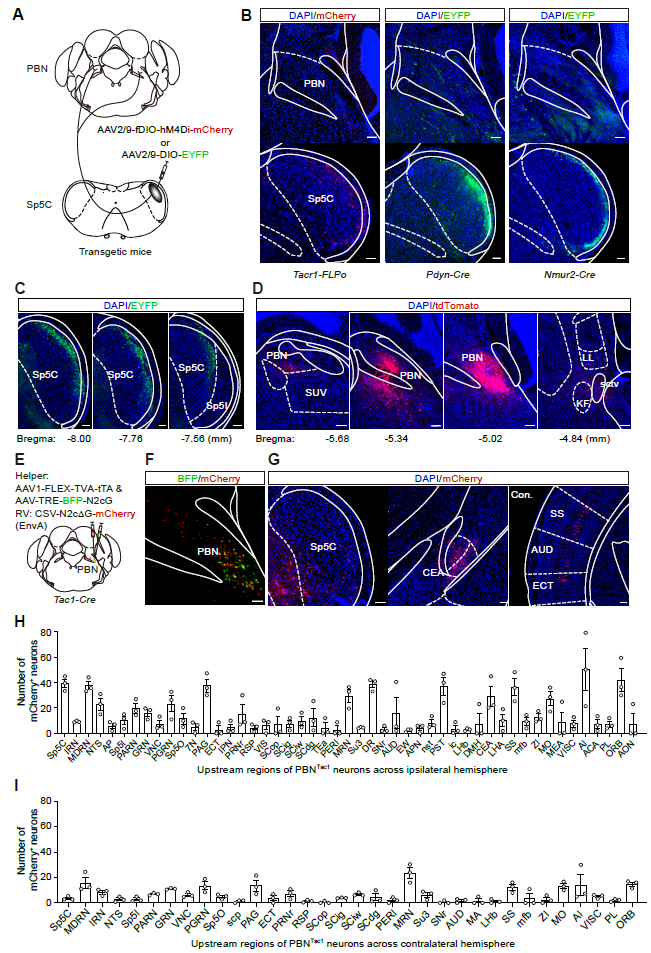


Fig. S5, related to Fig. 3. The major cell type of PBN-projecting Sp5C neurons is Tac1-positive. (A), A schematic for anterograde tracing in transgenic mice. (B), Images showing the distribution of EYFP/mCherry-labeled Sp5C neurons (bottom) and their axons in PBN (top) in *Tacr1-FLPo* mice (left), *Pdyn-Cre* mice (middle) and *Nmur2-Cre* mice (right). Red: mCherry, green: EYFP, blue: DAPI. Scale bar: 100 μm. (C), Images showing DIO-EYFP viral spread range in Sp5C of *Tac1-Cre* mice (related to Fig. 3E, 3F). (D), Images showing FLEX-tdTomato virus spread range in PBN of *Tac1-Cre* mice (related to Fig. 3G, 3H). (E), A schematic for rabies virus-based retrograde tracing. (F), Distribution pattern of BFP-positive neurons (pseudo color: green) and mCherry-labeled RV-positive neurons (red) in the right PBN. Scale bar: 100 μm. (G), Distribution pattern of mCherry-labeled RV-positive neurons (red) in the right Sp5C, the right CEA, and the left SS/AUD/ECT. Blue: DAPI. Scale bar: 100 μm. CEA: central amygdala nucleus, SS: somatosensory areas, AUD: auditory areas, ECT: ectorhinal cortex. (H), Average number of mCherry-positive neurons measured in each section of ipsilateral hemisphere. (I), Average number of mCherry-positive neurons measured in each section of contralateral hemisphere.


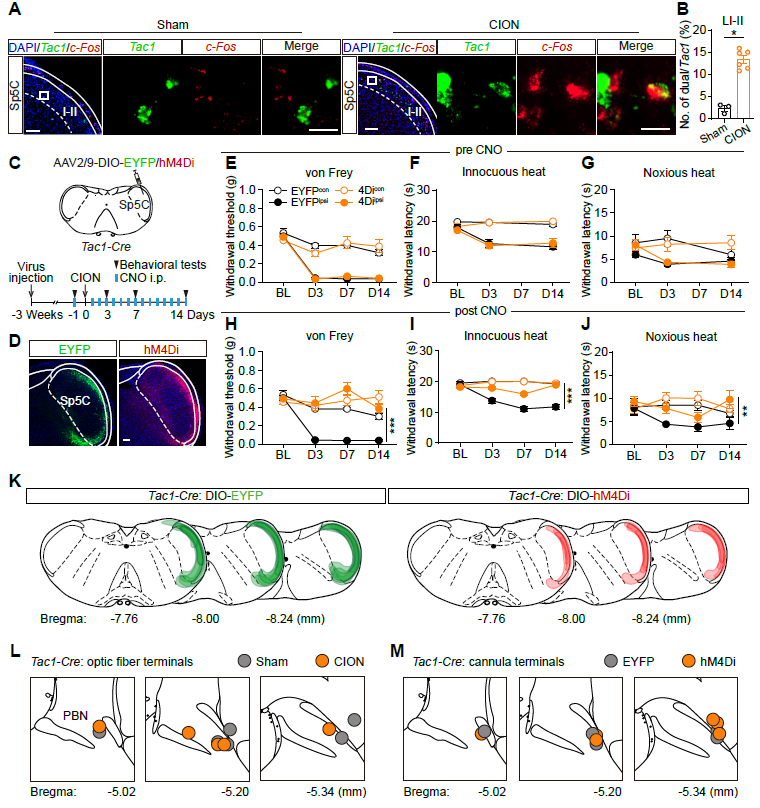


Fig. S6, related to Fig. 4. Sp5C^Tac1^ neurons play a critical role in CION-induced hypersensitivity. (A), Examples of RNAscope ISH results showing the gene expression distribution pattern of *c-Fos* (red) and *Tac1* (green) in the right Sp5C from Sham and CION groups on D14 after surgery. Blue: DAPI. Scale bar: 100 μm. Inset scale bar: 20 μm. (B), Ratio of dual-labeled to *Tac1*-expressing neurons in the right Sp5C of mice from both groups. **p* < 0.05, by Mann Whitney test. (C), A schematic and timeline for the experiment. (D), The images showing examples of virus expression pattern in EYFP and hM4Di groups of *Tac1-Cre* mice. Green: EYFP, red: hM4Di. Scale bar: 100 μm. (E-J), Withdrawal threshold (E, H, EYFP, n = 12; hM4Di, n = 11), and withdrawal latency of innocuous heat (F, I) and noxious heat (G, J) measured in mice from both groups. ***p* < 0.01, ****p* < 0.001, by two-way ANOVA (n = 6 for each group). (K), The brain atlases showed the expression pattern of DIO-EYFP and DIO-hM4Di-mCherry virus in Sp5C. (L), The brain atlases showing the location of optic fiber terminals in PBN of mice in Sham and CION groups (related to Fig. 4E-4K). Gray dot: optic fiber terminals in Sham group; orange dot: optic fiber terminals in CION group. (M), The brain atlases showing the location of cannula terminals in PBN of EYFP and hM4Di groups (related to Fig. 4L-4R). Gray dot: cannula terminals in EFYP group; orange dot: cannula terminals in hM4Di group.


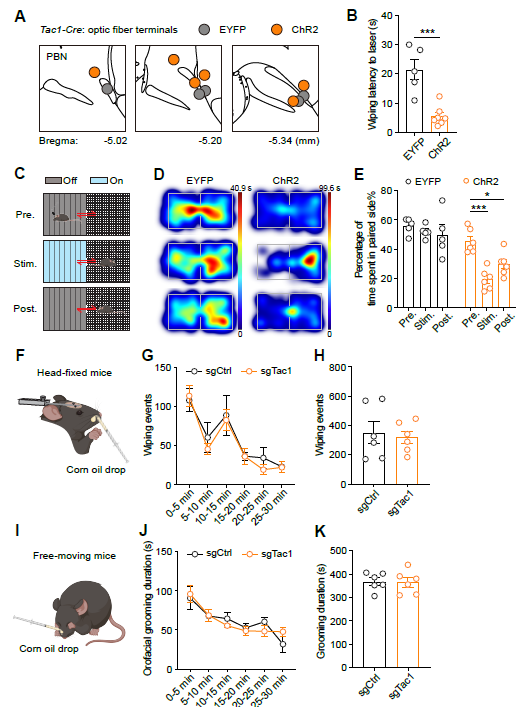


# Fig. S7, related to Fig. 4 PBN-projecting Sp5C^Tac1^ neurons contribute to orofacial pain-like behavior but not grooming behavior. (A), The brain atlases showing optic fiber terminals in PBN of *Tac1-Cre* mice for both groups (related to Fig. 4S-4V). Gray dot: optic fiber terminals in EYFP group; orange dot: optic fiber terminals in ChR2 group. (B), Latency of wiping behavior for mice in two groups during laser-on. ****p* < 0.001, by unpaired t test. (C), A schematic for real-time place aversion (RTPA) test of mice to laser-off (Pre. and Post.) and laser-on (Stim.) paired in the left side. Stim.: stimulation. (D), A heatmap showcase of time spent in chambers of mice in two groups. The blue-to-red scale shows increasing time spent in the zone. (E), Analysis of time spent in laser-paired side of mice in two groups at each stage of RTPA test. **p* < 0.05, ****p* < 0.001, by two-way ANOVA. (F), A schematic for orofacial grooming-like behavior test of head-fixed mice. Corn oil (100 μL) was dropped on the right side of whisker pad. (G), The time course of wiping events of mice in two groups counted in 5-min blocks across 30 min after oil administration. (H), Analysis of total wiping events of mice in two groups. (I), A schematic for orofacial grooming-like behavior test of freely moving mice. Corn oil (100 μL) was dropped on the top between both sides of whisker pad. (J), The time course of orofacial grooming duration of mice in two groups counted in 5-min blocks across 30 min after oil administration. (K), Analysis of total orofacial grooming duration of mice in two groups.


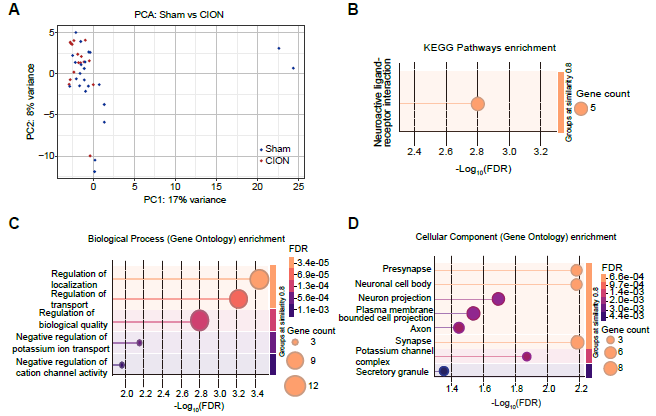


Fig. S8, related to Fig. 5. Key DEGs enrichment analysis of PBN-projecting Sp5C^Tac1^ neurons between Sham and CION groups. (A), Principal component analysis of sample gene expression levels (n = 25 for Sham; n = 26 for CION). (B), Biological Process (Gene Ontology) enrichment analysis for key DEGs between Sham and CION groups. (C), Cellular Component (Gene Ontology) enrichment analysis for key DEGs between two groups. (D), Kyoto Encyclopedia of Genes and Genomes (KEGG) enrichment analysis for key DEGs between two groups.


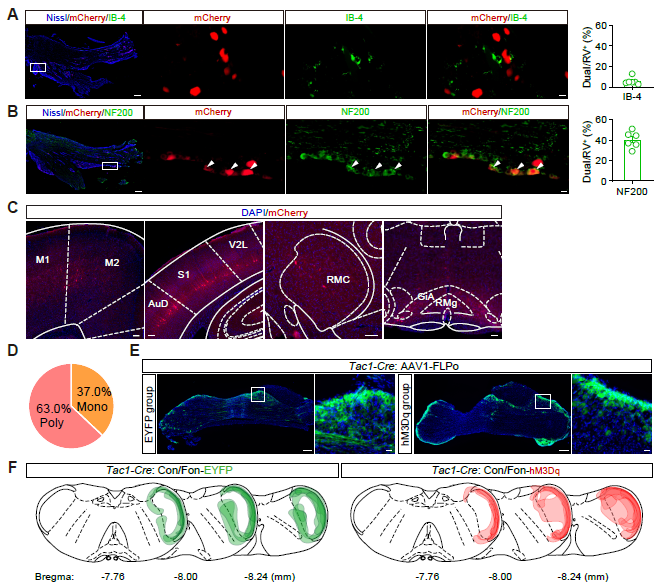


Fig. S9, related to Fig. 6. TG is one of upstream brain areas of PBN-projecting Sp5C^Tac1^ neurons contributes to orofacial pain-like behavior. (A-B), Distribution pattern of mCherry and IB-4- (A)/NF200- (B) positive neurons in right TG (left). Ratio of IB-4-/NF200-positive neurons in RV-expressed neurons (right). Red: mCherry, green: IB-4/NF200, blue: Nissl. Scale bar: 200 μm. Inset scale bar: 20 μm. (C), Examples of virus expression pattern in contralateral of M1, M2, V2L/S1/AuD, RMC and GiA/RMg. M1: primary motor cortex, M2: secondary motor cortex, V2L: lat area of secondary visual cortex, S1: primary somatosensory cortex, AuD: dorsal secondary auditory cortex, RMC: magnocellular part of red nucleus, GiA: alpha part of gigantocellular reticular nucleus, RMg: raphe magnus nucleus. Red: mCherry-labeled RV^+^ neurons, blue: DAPI. Scale bar: 100 μm. (D), Proportion of PBN-projecting Sp5C^Tac1^ neurons that displayed light-evoked monosynaptic (Mono.) or polysynaptic (Poly.) responses (n = 27 neurons from 12 mice). (E), Images showing virus expression pattern in right TG of mice from EYFP (left) and hM3Dq (right) group. Green: CTB-488 signals in TG neurons indicating AAV1-FLPo viral infection, blue: DAPI. Scale bar: 200 μm. Inset scale bar: 20 μm. (F), The brain atlases showed the expression pattern of Con/Fon-EYFP and Con/Fon-hM3Dq in Sp5C (related to Fig. 6L-6R).
